# Species Diversity With Comprehensive Annotations of Wood-Inhabiting Poroid and Corticioid Fungi in Uzbekistan

**DOI:** 10.3389/fmicb.2020.598321

**Published:** 2020-12-09

**Authors:** Yusufjon Gafforov, Alexander Ordynets, Ewald Langer, Manzura Yarasheva, Adriana de Mello Gugliotta, Dmitry Schigel, Lorenzo Pecoraro, Yu Zhou, Lei Cai, Li-Wei Zhou

**Affiliations:** ^1^Laboratory of Mycology, Institute of Botany, Academy of Sciences of the Republic of Uzbekistan, Tashkent, Uzbekistan; ^2^State Key Laboratory of Mycology, Institute of Microbiology, Chinese Academy of Sciences, Beijing, China; ^3^Department of Ecology, University of Kassel, Kassel, Germany; ^4^Institute of Applied Ecology, Chinese Academy of Sciences, Shenyang, China; ^5^Núcleo de Pesquisa em Micologia, Instituto de Botânica, São Paulo, Brazil; ^6^Department of Ecology and Botany, Andijan State University, Andijan, Uzbekistan; ^7^Tashkent State Agrarian University, Tashkent, Uzbekistan; ^8^Faculty of Biological and Environmental Sciences, University of Helsinki, Helsinki, Finland; ^9^Global Biodiversity Information Facility (GBIF), Secretariat, Universitetsparken, Copenhagen, Denmark; ^10^School of Pharmaceutical Science and Technology, Health Sciences Platform, Tianjin University, Tianjin, China; ^11^Graduate School of Geography, Clark University, Worcester, MA, United States

**Keywords:** Basidiomycota, Central Asia, distribution, substrate preferences, taxonomic diversity, GIS mapping

## Abstract

Uzbekistan, located in Central Asia, harbors high diversity of woody plants. Diversity of wood-inhabiting fungi in the country, however, remained poorly known. This study summarizes the wood-inhabiting basidiomycte fungi (poroid and corticoid fungi plus similar taxa such as *Merismodes, Phellodon*, and *Sarcodon*) (Agaricomycetes, Basidiomycota) that have been found in Uzbekistan from 1950 to 2020. This work is based on 790 fungal occurrence records: 185 from recently collected specimens, 101 from herbarium specimens made by earlier collectors, and 504 from literature-based records. All data were deposited as a species occurrence record dataset in the Global Biodiversity Information Facility and also summarized in the form of an annotated checklist in this paper. All 286 available specimens were morphologically examined. For 138 specimens, the 114 ITS and 85 LSU nrDNA sequences were newly sequenced and used for phylogenetic analysis. In total, we confirm the presence of 153 species of wood-inhabiting poroid and corticioid fungi in Uzbekistan, of which 31 species are reported for the first time in Uzbekistan, including 19 that are also new to Central Asia. These 153 fungal species inhabit 100 host species from 42 genera of 23 families. Polyporales and Hymenochaetales are the most recorded fungal orders and are most widely distributed around the study area. This study provides the first comprehensively updated and annotated the checklist of wood-inhabiting poroid and corticioid fungi in Uzbekistan. Such study should be expanded to other countries to further clarify species diversity of wood-inhabiting fungi around Central Asia.

## Introduction

Central Asia is a biological crossroads at the most westerly part of the Himalayan range and supports both Palearctic species and others representative of more southerly subtropical latitudes. The peculiarity of fauna and flora is due to its mixed characters: Indo-Himalayan, Mongolian, Eurasian, and Mediterranean species are present (Anonymous, 2001). Uzbekistan in the heart of Central Asia has a diversity of habitats that are globally and regionally important in ecological functions. The varying landscapes of high mountain ranges, wide steppes, deserts, and riparian wetlands in Uzbekistan result in a high diversity of habitats. The mountain areas occupy 15% of the territory of Uzbekistan. Biodiversity of Uzbekistan includes more than 27,000 species, including over 15,000 animal species; plants, algae, and fungi total about 11,000 species (Anonymous, 1998). The flora of Uzbekistan includes 4500 species of vascular plants, of which about 400 species are endemic, rare, and relict. Many of the animals and higher plants are included in the Red List of the International Union for Conservation of Nature (IUCN) and the Red Book of the Republic of Uzbekistan.

In contrast to the great number of publications dealing with the flora, limited studies document the fungi in Uzbekistan and Central Asia in general (Gafforov, 2017; Gafforov et al., 2017). Current surveys in areas of high plant endemism, such as tropical and subtropical regions, are actually showing an even higher ratio of fungal to plant diversity and uncovering an extraordinary number of endemic fungi (e.g., Crous et al., 2006; Mueller and Schmit, 2007; Schmit and Mueller, 2007; Aime and Brearley, 2012; Hawksworth, 2012). Mountains of the Central Asia Biodiversity Hotspot consist of two major mountain systems, the Pamir and the Tien Shan. Both belong to the most diverse regions in the world with respect to fauna and flora and are regarded as areas of occurrence of many endemic, relict, and endangered species. Therefore, diverse and regionally limited fungi are expected to exist in the region. However, while knowledge of fungal diversity is developing rapidly in some areas of the world, data on the fungi in Central Asia are severely limited (Gafforov et al., 2017; Antonelli et al., 2020; Cheek et al., 2020): the current knowledge of Uzbekistan fungal biodiversity status and even a rough estimate of the number of fungal species in countries of Central Asia is unavailable. This knowledge gap has significantly impeded understanding the role of the region in biogeographic history of Asia and prevented conservation efforts in the region.

Fungi are essential components of ecosystems and are both directly and indirectly important for human cultures. Various fungal species are key symbionts of trees enabling the survival of the latter in the arid areas (Varma, 1995; Stutz et al., 2000). Fungal names used in other regional floras have often been applied to fungi in Uzbekistan. However, the Uzbek fungi often represent new, unrelated species as was shown for Uzbekistan ascomycetous microfungi (Solieva and Gafforov, 2001, 2002; Gafforov and Hoshino, 2015; Gafforov, 2002, 2010, 2015, 2016a,b; Gafforov and Rakhimov, 2017; Gafforov et al., 2019; Wanasinghe et al., 2017, 2018a,b; Samarakoon et al., 2018; Pem et al., 2018, 2019a,b; Hyde et al., 2019, 2020; Yuan et al., 2020). Basidiomycetous fungi have received even less attention than ascomycetous microfungi (Gafforov, 2014; Gafforov et al., 2014, 2017).

Among the basidiomycetous macrofungi, especially those with poroid fertile surface of fruiting bodies (poroid fungi) and corticioid fertile surface (corticioid fungi) play several essential roles in forest ecosystems (Swift, 1982). Most of these fungi are saprobes causing brown or white wood rot, whereas some of them form ectomycorrhizae with woody plants. Therefore, they play an important function in nutrient cycling and soil formation (Soudzilovskaia et al., 2019). Some of them are also known to be serious pathogenic disease agents of ecologically and economically important coniferous and deciduous woody plants. Regardless of the relationship, wood-inhabiting basidiomycetous fungi are often treated as a single research object by both taxonomists and ecologists.

The first mycological investigations on wood-inhabiting fungi in Uzbekistan were started by Sinadskiy and Bondartseva in 1950, who reported 21 polypore species (Sinadskiy and Bodartseva, 1956, 1960; Kleyner, 1958; Panfilova and Gaponenko, 1963; Gaponenko, 1965; Sinadskiy, 1968). The first study specifically in state reserves of Uzbekistan listed 71 polypore species (Baltaeva, 1992, 1993). In the study of macrofungi of Fergana valley (Andijan, Fergana and Namangan Provinces), Tashkent Province, 25 species of poroid and corticioid fungi were reported (Khalikova, 1989; Iminova, 2009). However, the fungal species recorded in these studies were identified solely by morphological characters and no specimen was preserved, which makes the reassessment of taxonomic affiliation of these records impossible.

Recent developments in DNA sequencing have revolutionized identification and systematics of fungi. This has rapidly advanced the mycological communities’ ability to document fungal biodiversity, distribution, ecological preferences, and biogeographic history (e.g., Hattori et al., 2012; Ovaskainen et al., 2013; Tsykun et al., 2013; Tedersoo et al., 2014). DNA barcodes can facilitate taxonomic research by increasing the ability to matching individuals regardless of the fruiting body, identifying specimens with morphological diagnostic characters either subtle, difficult to visualize, or absent, as well as reassessing intraspecific polymorphisms. With the aid of DNA sequences, research on the wood-inhabiting basidiomycetous fungi during the last decade has yielded some species previously unknown in Uzbekistan, as well as some species new to science (Gafforov, 2014; Gafforov et al., 2014, 2017; Yuan et al., 2017, 2020; Kan et al., 2017). Moreover, the first fungal checklist of the corticioid genus *Hyphodontia* from Central Asia was published (Gafforov et al., 2017). However, despite these steps forward, comprehensive information of the wood-inhabiting poroid and corticioid fungi is still unavailable in Uzbekistan.

On the basis of our own collections, literatures, and herbarium data reassessments, the present study aimed to recognize species diversity of wood-inhabiting poroid and corticioid fungi (plus similar taxa such as *Merismodes, Phellodon* and *Sarcodon*) in Uzbekistan from morphological and, where possible, phylogenetic perspectives, and also to provide comprehensive annotations for these species including host, substratum, distribution, and occurring frequency.

## Materials and Methods

### Vegetation and Climate of the Study Area

**FIGURE 1:**
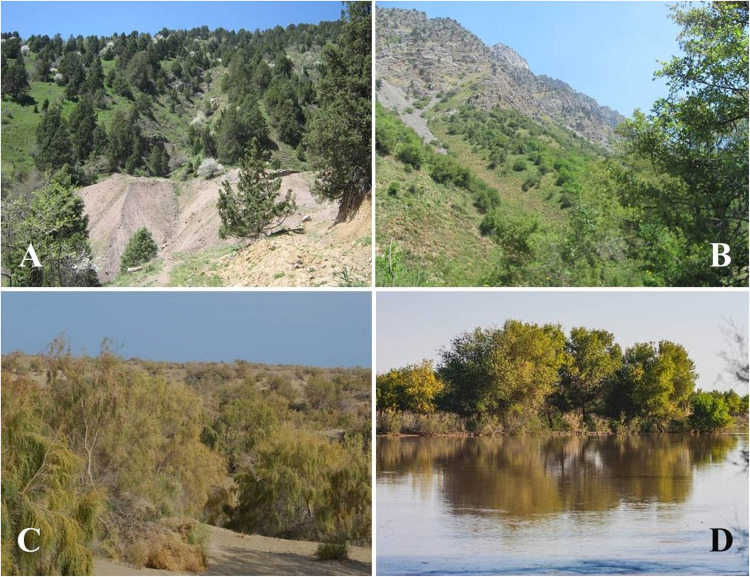
Forest types in study area. **(A)** Mountain juniper forests. **(B)** Wild fruit tree forests in mountain. **(C)** Desert saxaul (*Haloxylon* spp.) forests. **(D)** Tugai Forests (Photo: Yusufjon Gafforov).

The Uzbekistan territory falls in the flora of Central Asian botanical region within the larger temperate Asia floral geographic region according to the World Geographical Scheme for Recording Plant Distributions’ system (Brummitt, 2001). The main ecological forest types in Uzbekistan are mountain, desert, and flood-plain forests (Figure 1). The majority of Uzbekistan forests are xerophytic open woodlands of deciduous trees and shrubs, constituting about 7.3% of the territory (Botman, 2009). These forests play an important role in the protection and prevention from environmental degradation, particularly land degradation and natural disasters, and also in the conservation of biodiversity and preservation of water quality.

Uzbekistan has a continental climate with hot and dry summers and cold winters. Summer temperature often surpasses 40°C (104°F), and winter average temperature is about −2°C (28°F) but may fall as low as −40°C (−40°F). Most parts of the country are arid with average annual rainfall amounting to between 100 and 200 mm (3.9 and 7.9 in) and occurring mostly in winter and spring. Between July and September, little precipitation falls, essentially stopping the growth of vegetation during that period (Klein Tank et al., 2006; Lioubimtseva and Henebry, 2009).

### Specimen Assembly

**FIGURE 2:**
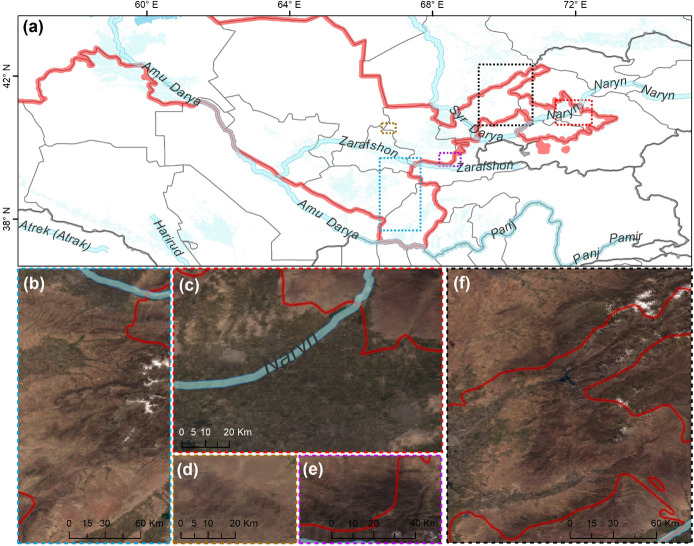
Map of **(a)** Uzbekistan labeled with five principal collecting areas shown in panels **(b–f)**. The background map in panels **(b–f)** is the combination of bands 1, 4, 3 displays and R, G, B surface reflectance image using 500-m MODIS data (MODS09A1) acquired in July 2019 over Uzbekistan (by Yu Zhou).

A total of 286 specimens of wood-inhabiting poroid and corticoid were examined. This includes 101 specimens from Mycological Herbarium of Estonian University of Life Sciences, Tartu, Estonia (TAAM); 3 specimens from Tashkent Mycological Herbarium, Institute of Botany of the Academy of Sciences of Uzbekistan, Tashkent (TASM); and 185 specimens from our own field surveys, which are deposited in TASM. Our own specimens were recently collected from Tashkent Botanical Garden (Tashkent city), Tashkent Province (Ugam-Chatkal State Nature National Park in Western Tien Shan Mountain), Jizzakh Province (Zaamin National Nature Park, Zaamin State Reserve in Turkestan range and Nurata State Reserve in Nurata range of Pamir-Alay), Surxondaryo Province (Baysun and Husar ranges in Pamir-Alay Mountains), and Fergana Valley (Namangan Province) (Figure 2). In addition, we reviewed 504 records of Uzbekistan fungi published between 1950 and 2020.

### Morphological Study

Morphological characters were described based on fresh and dried fruiting bodies. Microscopic characters of fruiting bodies were observed on dried specimens at a magnification up to 1000 × with a Leica DM 1000 (Tokyo, Japan) microscopes in 5% aqueous KOH plus 1% phloxine, Melzer’s reagent for amyloid or dextrinoid reactions, cotton blue in lactic acid for cyanophily, and 1% aqueous cresyl blue for metachromatism (Hawksworth et al., 1995). Macromorphological characters of fruiting bodies and hymenophores were observed under a Leica M165 FC stereomicroscope. Scientific names, both of the fungi and the host plants, were checked for potential synonyms in the databases Index Fungorum (2020) and ThePlantList (2013), respectively. Species whose taxonomic placement is not established are listed under *incertae sedis*.

### DNA Extraction, Amplification, and Sequencing

Genomic DNA was extracted from the dried basidiocarps of herbarium materials using DNeasy Plant Mini Kit (Qiagen, Valencia, CA, United States), QIAamp DNA Micro Kit (Qiagen), and the Extract-N-Amp Plant PCR Kit (Sigma-Aldrich, St. Louis, MO, United States), following protocols from the manufacturers, and was diluted as a template for subsequent amplification. PCR amplification targeted the internal transcribed spacer (ITS) region of the ribosomal RNA gene (rRNA), the universal DNA barcode for identification of fungi (Schoch et al., 2012), and the nuclear large ribosomal subunit (nLSU) region. Amplification was carried out using the fungal-specific primer sets ITS1F/ITS4b (Gardes and Bruns, 1996) and ITS1/ITS4 (White et al., 1990) for the ITS region and LR0R and LR5 for the nLSU region (Vilgalys and Hester, 1990; Rehner and Samuels, 1994). Purified PCR products were sequenced using DNA ABI 3730 XL automated sequencers (Applied Biosystems) by Macrogen Inc. (Seoul, Korea), by Eurofins Genomics (Ebersberg, Germany), and by the Beijing Genomics Institute (Beijing, China). All newly generated sequences of poroid and corticoid species from Uzbekistan were submitted to GenBank (Table 1).

**TABLE 1:** Newly generated sequences and specimens used for the phylogenetic analyses.

Species	Specimens voucher	Host/geographic origin	GenBank accession no.		References
			ITS	LSU	
*Athelia arachnoidea*	CBS 105.18	Unknown/DE	MH854664	MH866181	Vu et al., 2019
	YG-G23	Fallen angiosperm/UZ	MT526279		This study
	YG/PS154	*Crataegus* sp./UZ		MT524543	This study
	YG1111	*Lonicera* sp./UZ		MT524544	This study
*Aurantiporus fissilis*	BRNM 699803	*Populus tremula*/CZ	HQ728292	HQ729002	Tomšovskyì, 2012
	YG/bot3	*Juglans regia*/UZ	MT526280	MT524545	This study
*Bjerkandera adusta*	CBS 371.52	Unknown/JP	MH857085	MH868620	Vu et al., 2019
	YG/bot23a	Unknown wood/UZ	MT526285	MT524549	This study
	YG-G41	Dead stump of *Prunus armeniaca*/UZ	MT526286		This study
	YG-O1	Fallen trunk of *Populus* sp./UZ	MT526284	MT524548	This study
	YG/PS172	*Juglans regia*/UZ	MT526281	MT524546	This study
	YG/PS183	Dried trunk of angiosperm/UZ	MT526283	MT524547	This study
	YG012	*Juglans regia*/UZ	MT526282		This study
*Byssomerulius corium*	CBS 836.72	Unknown/NL	MH860620		Vu et al., 2019
	YG-G21	Angiosperm fallen branch/UZ	MT526287	MT524550	This study
	YG-X3	Dried branch of *Prunus vulgaris*/UZ	MT526288	MT524551	This study
*Cerrena unicolor*	FD 299	Dead standing hardwood/US	KP135304	KP135209	Floudas and Hibbett, 2015
	YG-G28	Dried fallen stem of angiosperm/UZ	MT526292		This study
	YG/PS79	*Acer tataricum* subsp. *semenovii*/UZ	MT526291	MT524554	This study
	YG003	*Crataegus pseudoheterophylla* subsp. *turkestanica*/UZ	MT526289	MT524552	This study
	YG18	*Acer tataricum* subsp. *semenovii*/UZ	MT526290	MT524553	This study
*Ceriporiopsis gilvescens*	YG046	Dried angiosperm wood/UZ		MT524555	This study
	YG049	Rotten trunk of wood/UZ	MT526293		This study
	Yuan 2752	Unknown/CN	KF845953	KF845946	Zhao and Cui, 2014
*Flavidoporia pulverulenta*	BR3450	*Picea abies*/FR	JQ700280	JQ700280	Spirin et al., 2013
	YG/PS167	*Salix* sp./UZ	MT526295	MT524557	This study
	YG1110	*Salix alba*/UZ	MT526294	MT524556	This study
*Fomes fomentarius*	Cui 5769	Unknown/CN	KX885072	KX832056	Direct submission
	YG/bot2	*Populus* sp./UZ	MT526299	MT524558	This study
	YG/bot4	Decaying trunk of angiosperm/UZ	MT526300		This study
	YG/Un2	*Juglans regia*/UZ	MT526302		This study
	YG/PS174	*Juglans regia/*UZ	MT526301	MT524559	This study
	YG014	*Juglans regia*/UZ	MT526298		This study
	YG-60	Unknown wood/UZ	MT526296		This study
	YG-70	Unknown wood/UZ	MT526297		This study
*Ganoderma adspersum*	FGA1	*Pterocarya fraxinifolia*/IT	AM269771	AM269829	Guglielmo et al., 2007
	YG/bot24	*Acer saccharum*/UZ	MT526303	MT524560	This study
*Ganoderma resinaceum*	MFLU 19-2153	*Albizia mollis*/CN	MN398315	MN398328	Direct submission
	YG-X4	*Salix* sp./UZ	MT526304	MT524561	This study
*Gloeophyllum abietinum*	Dai 3595	*Pinus* sp./CN	JX524620	KC782732	He et al., 2014
	TAAM127397	Trunk of *Juniperus* sp./UZ	MT526305		This study
*Hyphoderma setigerum*	FP150263	Unknown/JM	GQ409528	KX065980	Direct submission
*Hyphoderma* sp.	YG/PS133	*Betula* sp./UZ	MT526306		This study
*Hyphodontia zhixiangii*	LWZ 20160909-4	*Juniperus* sp./UZ	NR154098		Kan et al., 2017
	YG1098	Unknown angiosperm branch/UZ	MT526308		This study
	YG1104	Fallen angiosperm branches/UZ	MT526307	MT524562	This study
*Inonotus hispidus*	S45	*Vitis vinifera*/ES	EU282482	EU282484	González et al., 2009
	YG/AG1	*Morus alba*/UZ		MT524564	This study
	YG/bot1	Dried trunk angiosperm/UZ		MT524565	This study
	YG/PS148	*Malus sieversii*/UZ	MT526310	MT524567	This study
	YG/PS156	*Pinus* sp./UZ	MT526309	MT524563	This study
	YG/PS157	*Pinus* sp./UZ		MT524566	This study
*Lentinus tigrinus*	CBS 249.39	Unknown/Yugoslavia	MH856001	MH867501	Vu et al., 2019
	YG-J7	Dried strum of angiosperm/UZ	MT526312	MT524569	This study
	YG/PS162	*Malus domestica*/UZ	MT526311	MT524568	This study
	TAAM094847	*Acer* tree trunk/UZ	MT526313		This study
	TAAM094857	*Prunus armeniaca*/UZ	MT526314		This study
*Lenzites warnieri*	CIRM-BRFM 972	Unknown/FR	GU731567		Direct submission
	TAAM126870	*Populus nigra*/UZ	MT526315		This study
*Lyomyces erastii*	RM21	Unknown shrub/UZ	MT526316		This study
	162SAMHYP	*Sambucus racemosa*/FI	JX857801		Direct submission
*Neoantrodiella* sp.	TAAM104307	*Juniperus polycarpos* var. *seravschanica*/UZ	MT526317		This study
*Neoantrodiella gypsea*	Yuan 5589	Unknown/CN	KT203292	KT203313	Ariyawansa et al., 2015
*Peniophora cinerea*	He 3725	Unknown/CN	MK588769	MK588809	Direct submission
	YG058	*Juglans regia*/UZ	MT526318		This study
*Peniophora incarnata*	CBS 430.72	Unknown/NL	MH860518	MH872230	Vu et al., 2019
	YG/PS84	Fallen stem of deciduous wood/UZ	MT526319	MT524570	This study
*Peniophorella praetermissa*	GEL2182	*Betula* sp./NO	AY854081	AY700185	Direct submission
	YG-G16	*Juglans regia*/UZ	MT526320	MT524571	This study
	YG-G37	Fallen unknown angiosperm/UZ	MT526321		This study
	YG-G40	Fallen unknown angiosperm/UZ	MT526322		This study
*Phellinus betulinus*	DVB-Betula	*Betula nigra*/US	KU139151	KU139246	Brazee, 2015
	TAAM104436	*Betula tianschanica*/UZ	MT526323		This study
*Phellinus pomaceus*	CBS 171.32	Unknown/GB	AY558652	MH866714	Jeong et al., 2005; Vu et al., 2019
	TAAM126269	*Celtis australis* subsp. *caucasica*/UZ	MT526324	MT524572	This study
	TASM582	*Lonicera* sp./UZ	MT526326		This study
	YG/PS3X	*Cerasus tianshanica*/UZ	MT526328	MT524576	This study
	YG009	*Prunus dulcis*/UZ	MT526325	MT524574	This study
	YG/PS82	*Prunus* sp./UZ	MT526333		This study
	YG/S1	*Salix* sp./UZ		MT524573	This study
	YG51-ph	*Prunus* sp./UZ	MT526331		This study
	YG028	*Cerasus tianshanica*/UZ	MT526327	MT524575	This study
	YG052	*Prunus cerasifera*/UZ	MT526329		This study
	YG28	*Prunus mahaleb*/UZ	MT526330	MT524577	This study
	YG337	*Prunus cerasifera*/UZ	MT526332		This study
	YG1102	*Prunus* sp./UZ	MT526334		This study
*Phlebia bresadolae*	MG291	*Acer monspessulanum*/IR	KU213584		Ghobad-Nejhad and Langer, 2016
*Phlebia cf. bresadolae*	RLG10795s	Unknown/US	KY948785	KY948857	Justo et al., 2017
	YG/PS89	Unknown woody plants branch/UZ	MT526336	MT524578	This study
	YG/PS189	Fallen branch of angiosperm/UZ	MT526335		This study
*Phlebia* sp.	YG64	*Crataegus pseudoheterophylla* subsp. *turkestanica*/UZ	MT526337		This study
	YG326	Dead hardwood/UZ	MT526338	MT524579	This study
*Phlebia rufa*	MR 4280	Hardwood/US	KP135373	KX065989	Floudas and Hibbett, 2015; direct submission
	YG77	*Robinia pseudoacacia*/UZ	MT526339	MT524580	This study
*Phlebiella christiansenii*	KHL 11689	Unknown/FI	EU118659	EU118659	Larsson, 2007
	YG-G22	*Gleditsia triacanthos*/UZ	MT526340		This study
	YG-G26	Fallen angiosperm/UZ	MT526341		This study
	YG-G36	Dried stump of deciduous/UZ	MT526342		This study
*Phylloporia yuchengii*	YG-J5	*Populus* sp./UZ	MT526344	MT524584	This study
	YG-J10	*Morus alba*/UZ		MT524585	This study
	YG-J11	*Morus alba*/UZ		MT524586	This study
	YG033	Dead angiosperm trunk and stem/UZ		NG060132	Gafforov et al., 2014
	YG043	*Juglans regia*/UZ	MT526343	MT524581	This study
	YG343	*Prunus* sp./UZ		MT524582	This study
	YG1093	*Crataegus pseudoheterophylla* subsp. *turkestanica*/UZ		MT524583	This study
	YG1011	*Crataegus* sp./UZ		MT524587	This study
*Pilatoporus ibericus*	O 10811	*Pinus* sp./IT	KR605772	KR605711	Han et al., 2016
	YG-G24	Trunk of angiosperm/UZ	MT526345	MT524588	This study
*Radulomyces confluens*	K(M) 181613	Unknown/GB	MK953390	MK953401	Leal-Dutra et al., 2020
	YGcor-80	Strum of angiosperm/UZ	MT526346		This study
	YG-G43	Dead stump/UZ		MT524589	This study
*Rigidoporus ginkgonis*	Cui 5125	Rotten wood of Ginkgo/CN	KY131877	KY131933	Wu et al., 2017
	YG-G2	Strum of deciduous/UZ		MT524590	This study
	YG-G3	Strum of deciduous/UZ		MT524591	This study
	YG-G35	Decay branch of angiosperm/UZ	MT526347	MT524592	This study
*Sanghuangporus lonicerinus*	Dai 17095	*Lonicera* sp./UZ	MF772787	MF772806	Zhu et al., 2019
	TAAM0104264	*Lonicera* sp./UZ	MT526352	MT524597	This study
	TAAM127578	Dry trunk of deciduous trunk/UZ	MT526353	MT524598	This study
	YG/PS92	*Lonicera* sp./UZ	MT526348		This study
	YG/PS129	*Lonicera* sp./UZ	MT526349	MT524594	This study
	YG/Un1	*Acer tataricum* subsp. *semenovii*/UZ	MT526350	MT524595	This study
	YG018	*Lonicera nummulariifolia*/UZ		MT524593	This study
	YG1112	*Lonicera* sp./UZ	MT526351	MT524596	This study
*Schizophyllum commune*	CBS 124811	Unknown	MH863418	MH874930	Vu et al., 2019
	YG-J2	*Populus* sp./UZ	MT526354	MT524599	This study
	YG/PS169	*Juglans regia*/UZ	MT526355	MT524600	This study
*Stereum hirsutum*	CBS 930.70	Unknown/UZ	MH860009	MH871796	Vu et al., 2019
	TAAM104393	Deciduous tree trunk/UZ	MT526367		This study
	TAAM126291	*Populus alba*/UZ	MT526368	MT524610	This study
	YG-G12	*Fraxinus excelsior*/UZ		MT524601	This study
	YG-G15	*Quercus* sp./UZ	MT526362	MT524605	This study
	YG/PS135	*Juglans regia*/UZ	MT526363	MT524606	This study
	YG/PS176	*Juglans regia*/UZ	MT526364	MT524607	This study
	YG51	*Acer* sp./UZ	MT526356	MT524602	This study
	YG030	*Acer tataricum* subsp. *semenovii*/UZ	MT526357		This study
	YG048	Unknown dried wood/UZ	MT526358		This study
	YG056	Unknown decay wood/UZ	MT526359	MT524603	This study
	YG057	*Acer tataricum* subsp. *semenovii*/UZ	MT526360		This study
	YG320	*Salix alba*/UZ	MT526361	MT524604	This study
	YG1091	Unknown woody branch/UZ	MT526365	MT524608	This study
	YG1092	Fallen trunk of angiosperm wood/UZ	MT526366		This study
	YG3.04.13	Dead fallen trunk of angiosperm/UZ		MT524609	This study
*Subantrodia uzbekistanica*	Dai 17105	*Juniperus polycarpos* var. *seravschanica*/UZ	KX958183	KX958187	Yuan et al., 2017
	YG1100	Unknown woody branches/UZ	MT526370		This study
	YG1107	*Juniperus polycarpos* var. *seravschanica*/UZ	MT526371		This study
*Trametes hirsuta*	CBS 282.73	Unknown/DE	MH860685	MH872390	Vu et al., 2019
	RM44	Died angiosperm strum/UZ	MT526381		This study
	TAAM104394	*Juglans regia*/UZ	MT526369		This study
	YG/Ch40	Stem of angiosperm tree/UZ	MT526382	MT524614	This study
	YG/PS128	Unidentified angiosperm stem/UZ	MT526378	MT524612	This study
	YG/PS138	*Prunus* sp./UZ	MT526379		This study
	YG/PS168	*Juglans regia*/UZ	MT526380	MT524612	This study
	YG004	*Prunus armeniaca*/UZ	MT526372		This study
	YG007	*Prunus vulgaris*/UZ	MT526373		This study
	YG037	*Juglans regia*/UZ	MT526374		This study
	YG055	Unknown decay wood/UZ	MT526375		This study
	YG073	Dried unknown woody trunk/UZ	MT526376		This study
	YG314	*Prunus* sp./UZ	MT526377	MT524611	This study
*Trametes trogii*	Dai 11246	Unknown/CN	KC867380	KC867451	Li et al., 2016
	TAAM189940	unidentified wood/UZ	MT526387	MT524621	This study
	YG/bot23b	Fallen deciduous branch/UZ	MT534628		This study
	YG-G14	Unknown woody branches/UZ	MT534629	MT534627	This study
	YG-G17	Unknown strum of angiosperm/UZ	MT526383	MT524615	This study
	YG-G18	Unknown strum of angiosperm/UZ	MT526384	MT524616	This study
	YG-G19	Unknown strum of angiosperm/UZ		MT524617	This study
	YG-GX1	*Populus* sp./UZ	MT526388		This study
	YG/PS2X	Dried on *Salix* sp./UZ		MT524622	This study
	YG-J4	*Populus nigra*/UZ	MT526385	MT524619	This study
	YG-J6	*Populus nigra*/UZ	MT526386	MT524620	This study
	YG-JX4	*Populus* sp./UZ		MT524623	This study
	YG-N7	*Populus alba*/UZ		MT524618	This study
*Trametes versicolor*	Cui 9310	Unknown/CN	KC848266	KC848351	Direct submission
	YG-J3	*Prunus* sp./UZ	MT526389	MT524624	This study
	YG/PS170	*Juglans regia*/UZ		MT524625	This study
*Trametes villosa*	CBS 334.49	Unknown/AR	MH856545	MH868069	Vu et al., 2019
	YG/AG11	Dried stem of angiosperm/UZ	MT526390		This study
*Vuilleminia comedens*	CBS 428.72	Unknown/NL	MH860516	MH872229	Vu et al., 2019
*Vuilleminia* sp.	TAAM104410	*Lonicera* sp./UZ		MT524626	This study

### Phylogenetic Analyses

After a preliminary BLAST search, 40 sequences related to those from Uzbekistan specimens were downloaded from GenBank to assist species identification (Table 1). The datasets of ITS and nLSU regions were separately aligned using MAFFT 7.110 (Katoh and Standley, 2013) under the G-INI-i option (Katoh et al., 2005) and then the two alignments were concatenated. The concatenated alignment, deposited in TreeBASE^1^ (accession number S26575), was subjected to an estimation of the best-fit evolutionary model using jModelTest (Guindon and Gascuel, 2003; Posada, 2008) with calculation under Akaike information criterion. Following this model, maximum likelihood (ML) and Bayesian inference (BI) methods were employed for phylogenetic analyses. The ML method was conducted using raxmlGUI 1.2 (Stamatakis, 2006; Silvestro and Michalak, 2012) with calculation of bootstrap (BS) replicates under the auto FC option (Pattengale et al., 2010). The BI method was conducted using MrBayes 3.2 (Ronquist et al., 2012). Two independent runs were employed. Each run had four chains and started from random trees. Trees were sampled every 1000th generation, of which the first 25% were removed and the other 75% were used for constructing a 50% majority consensus tree and calculating Bayesian posterior probabilities (BPPs). Tracer 1.5^2^ was used to determine chain convergence. iTOL was used to visualize the tree to a circular form (Letunic and Bork, 2019).

### GBIF Occurrence Dataset and Checklist Preparation

The occurrence data of wood-inhabiting poroid and corticioid fungi was extracted from 504 records in 19 publications as well as 185 records of our own recent collections in field surveys and 101 herbarium specimens from TAAM and TASM. All but collection data from TAAM (which are already displayed in GBIF) were formatted according to the Darwin Core Standard^3^ and published as an occurrence dataset (Gafforov and Ordynets, 2020, ^4^ alternative identifier^5^). When compiling the annotated species checklist for this paper, for the sake of conciseness, all occurrence records were linked to 50 localities that are listed in the study.

### GIS Data Processing

A point distribution map of fungal orders was produced using the ArcGIS 10.7 desktop software (ArcGIS Desktop, 2020). A GPS navigation device and Google Earth software^6^ (2020) were used for geo-referencing all available occurrence data of wood-inhabiting poroid and corticioid fungi in the study sites. A WGS84 geographic coordinate system was used as a reference datum. The land cover data were adapted from the 500-m Moderate Resolution Imaging Spectroradiometer (MODIS) land cover product (MCD12Q1; Friedl et al., 2002) which has 17 IGBP classes, including water, evergreen needleleaf forests (ENF), evergreen broadleaf forests (EBF), deciduous needleleaf forests (DNF), deciduous broadleaf forests (DBF), mixed forests (MF), closed shrublands (CSH), open shrublands (OSH), woody savannas (WSA), savannas (SAV), grasslands (GRA), permanent wetlands, cropland (CRO), urban and built-up, cropland and natural vegetation mosaics (CNM), snow and ice, and barren. Considering the spatial distribution of irrigated and cultivated croplands, we further integrated these two classes from Klein et al. (2012). Data for roads, rivers, lake centerlines, and country boundaries were downloaded from the Natural Earth database (Natural Earth, 2020).

## Results

### Phylogenetic Placement of Collections of Poroid and Corticoid Fungi From Uzbekistan

**FIGURE 3:**
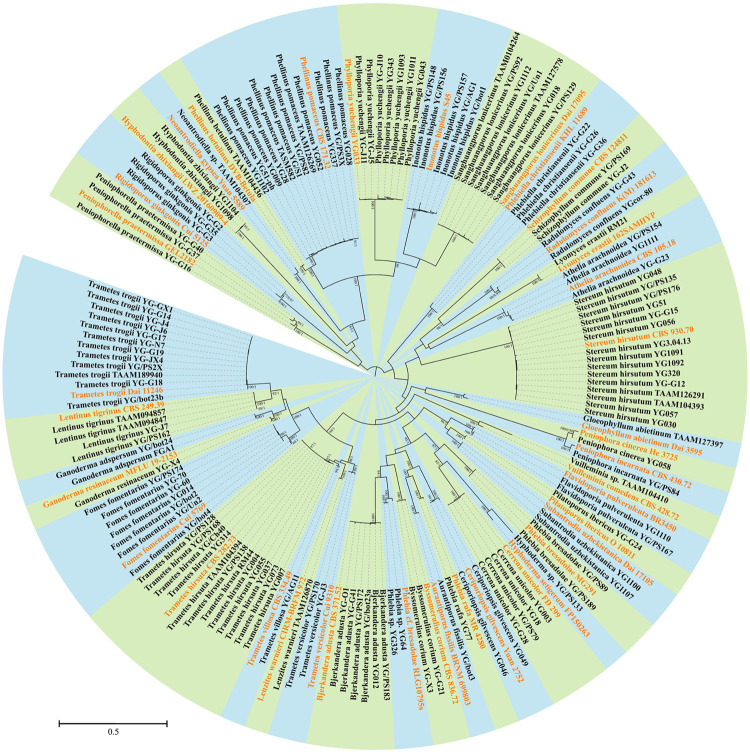
Phylogenetic position of newly sequenced Uzbekistan specimens of wood-inhabiting poroid and corticioid fungi based on a combination of ITS and nLSU sequences. Maximum likelihood tree with bootstrap support values and posterior probabilities inferred from Bayesian analysis is shown. The background for each identified species or undescribed lineage along with its reference sequence is set as one of the alternately appearing blue and green colors. The sequences of collections labeled in orange are downloaded from GenBank, while those in black are generated in this study.

In addition to morphological characters, DNA sequences were used to identify certain specimens. A total of 114 ITS and 85 LSU sequences from 138 specimens representing 40 species were newly generated for this study, and submitted to GenBank (Table 1). The alignment used for phylogenetic analysis included 178 collections (Table 1). The best-fit evolutionary model for this alignment was estimated as GTR + I + G. In the ML method, the BS search stopped after 250 replicates. In the BI method, all chains were converged after 6 million generations, where the average standard deviation of split frequencies is 0.006815, the estimated sample sizes of all parameters are above 700, and the potential scale reduction factor approaches 1.0. Both phylogenetic methods generated congruent topologies in main lineages, and thus only the topology from the ML method is visualized in a circle form with BS and BPP at the nodes (Figure 3). From a phylogenetic perspective, 36 species were recovered and four potential new lineages representing members of *Hyphoderma, Neoantrodiella, Phlebia*, and *Vuilleminia* were identified from the newly sequenced specimens.

### Species Diversity of Wood-Inhabiting Poroid and Corticoid Fungi in Uzbekistan

**FIGURE 4:**
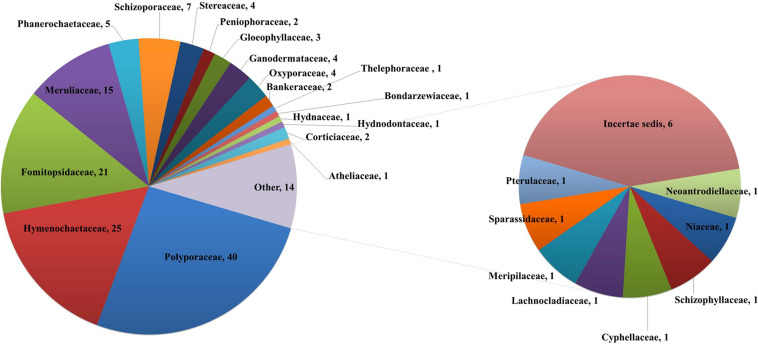
Taxonomic composition of wood-inhabiting poroid and corticoid fungi at a family level.

Taking literature information and morphological and phylogenetic evidence into consideration, we report 153 species of wood-inhabiting poroid and corticoid fungi including 149 described species and four single-specimen undescribed lineages belonging to 10 orders (Agaricales, Atheliales, Cantharellales, Corticiales, Gloeophyllales, Hymenochaetales, Russulales, Polyporales, Thelephorales and Trechisporales), 26 families, and 97 genera in Uzbekistan (Table 2 and Figure 4). Data on own specimens and extracted from literature records are accessible as an occurrence dataset (Gafforov and Ordynets, 2020,^4^ alternative identifier^5^). Among the 153 species, 31 are reported for the first time in Uzbekistan, including 19 also new to Central Asia. The orders represented by the most specimens are Polyporales (7 families, 59 genera, and 88 species) and Hymenochaetales (4; 20; 41). Together they contain 129 species or 84.3% of the total wood-inhabiting poroid and corticiod biota of Uzbekistan (Table 2). The most species-rich families are Polyporaceae (40 species in 25 genera), Hymenochaetaceae (25; 13), Fomitopsidaceae (21; 16), and Meruliaceae (15; 12) and contain 66 genera and 101 species that constitute 66% of the total poroid and corticoid species number. The genus with the highest number of recorded species are *Trametes* (9 species); *Inonotus* (5); *Ganoderma, Lentinus, Phellinus, Rigidoporus*, and *Stereum* (4 each); and *Antrodia, Cerioporus, Gloeophyllum, Fomitiporia, Hyphodontia, Lyomyces, Phlebia, Phylloporia, Postia*, and *Trichaptum* (3 each) that contain 64 species or 41.8%, and the other genera have one to two species (Table 2).

**TABLE 2:** Number of wood-inhabiting poroid and corticioid species in the most representative orders, families, and genera in the study area and proportion accounting for total species number.

ORDERS					FAMILIES				GENERA				
Order	Family	Genera	Spp.	%	Family	Genera	spp.	%	Genera		spp.		%
Polyporales	7	59	88	57.5	Polyporaceae	25	40	26.1	*Trametes*	9		5.87	
Hymenochaetales	4	20	41	26.8	Hymenochaetaceae	13	25	16.3	*Inonotus*	5		3.25	
Russulales	4	4	8	5.23	Fomitopsidaceae	16	21	13.7	*Antrodia*	4		2.61	
Agaricales	4	4	4	2.63	Meruliaceae	12	15	9.9	*Ganoderma*	4		2.61	
Thelephorales	2	3	3	1.97	Schizoporaceae	3	7	4.6	*Lentinus*	4		2.61	
Gloeophyllales	1	1	3	1.97	Phanerochaetaceae	4	5	3.26	*Phellinus*	4		2.61	
Corticiales	1	2	2	1.3	Ganodermataceae	1	4	2.61	*Stereum*	4		2.61	
Atheliales es	1	1	1	0.65	Oxyporaceae	1	4	2.61	*Cerioporus*	3		2.61	
Cantharellal	1	1	1	0.65	Stereaceae	1	4	2.61	*Hyphodontia*	3		2.61	
Trechisporales	1	1	1	0.65	Gloeophyllaceae	1	3	1.98	*Phylloporia*	3		2.61	
Subtotal	26	96	152	99.35	Subtotal	77	128	83.67	Subtotal	43		28.11	
with uncertain (1)	0	1	1	0.65	Other families (16)	20	25	16.33	Other genera (87)	110		71.89	
**Total**	**26**	**97**	**153**	**100%**	**26**	**97**	**153**	**100%**	**97**	**153**		**100%**	

### Annotated Checklist of Wood-Inhabiting Poroid and Corticioid Species in Uzbekistan

**FIGURE 5:**
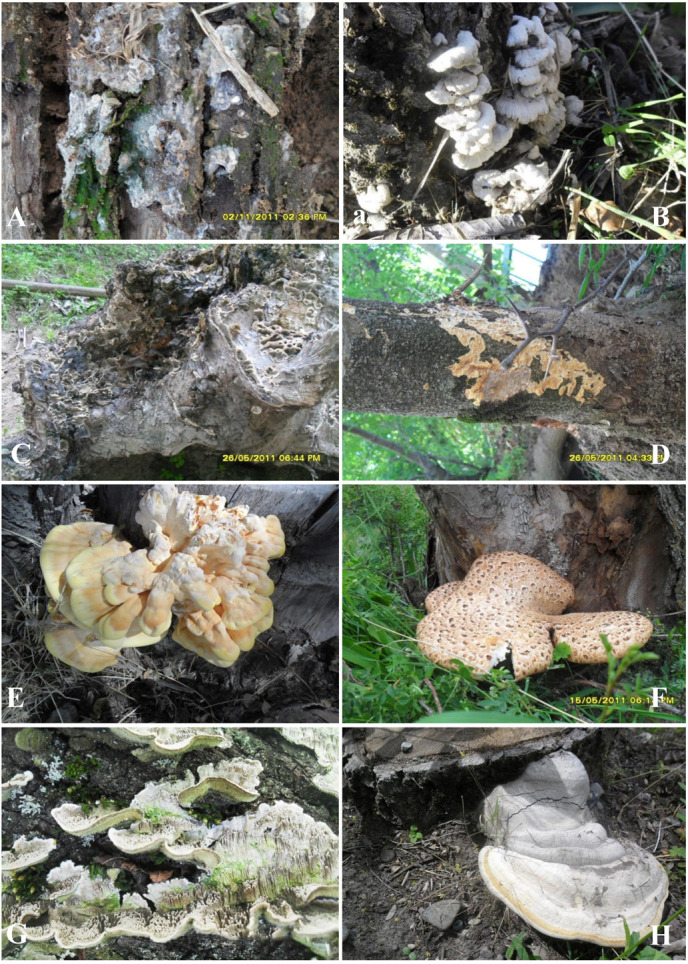
Basidiocarps in situ. **(A)** *Radulomyces confluens*; **(B)** *Schizophyllum commune*; **(C)** *Bjerkandera adusta*; **(D)** *Phlebia rufa*; **(E)** *Laetiporus sulphureus*; **(F)** *Cerioporus squamosus*; **(G)** *Cerrena unicolor*; **(H)** *Fomes fomentarius* (Photo: Yusufjon Gafforov).

**FIGURE 6:**
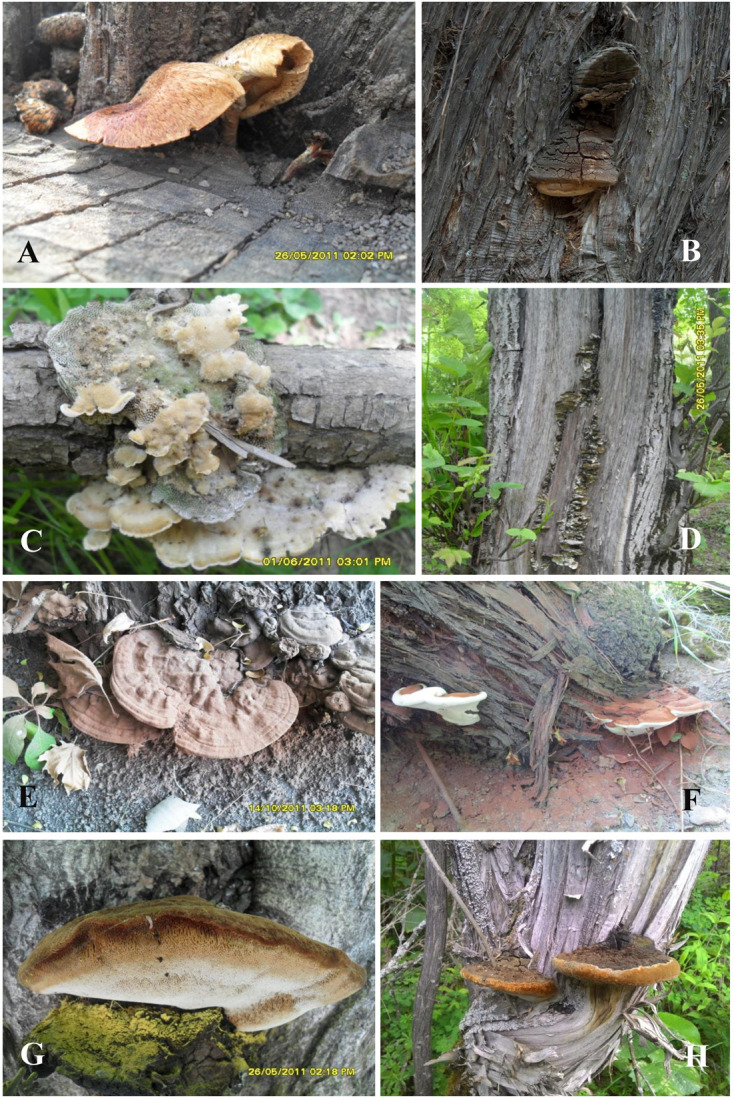
Basidiocarps *in situ* of **(A)** *Lentinus tigrinus*; **(B)** *Pyrofomes demidoffii*; **(C)** *Trametes hirsuta*; **(D)** *Trametes trogii*; **(E)** *Ganoderma adspersum*; **(F)** *Ganoderma applanatum*; **(G)** *Inonotus hispidus*; **(H)** *Phellinus igniarius* (Photo: Yusufjon Gafforov).

**FIGURE 7:**
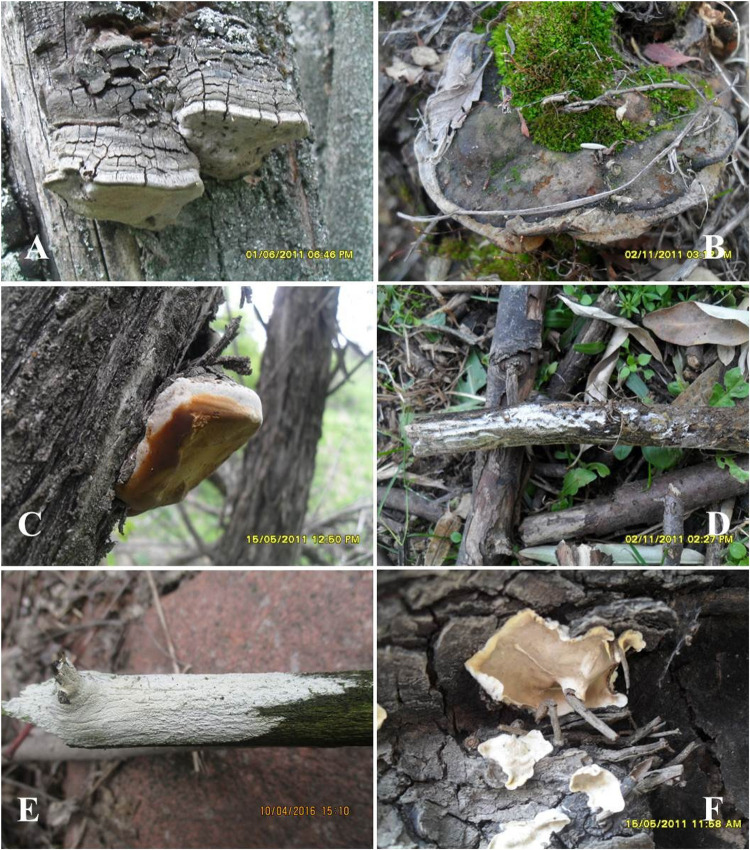
Basidiocarps *in situ* of **(A)** *Phellinus pomaceus*; **(B)** *Phylloporia yuchengii*; **(C)** *Sanghuangporus lonicerinus*; **(D)** *Lyomyces erastii*; **(E)** *Lyomyces sambuci*; **(F)** *Stereum hirsutum* (Photo: Yusufjon Gafforov).

The checklist of 153 species of wood-inhabiting poroid and corticioid fungi is arranged alphabetically by orders, family, and species. The currency sign (¤) indicates potentially new species to science and asterisk (^∗^) denotes new fungal records to Central Asia and thus to Uzbekistan, while the new fungal records to Uzbekistan but not to Central Asia is indicated by a number sign (#). A filled circle (•) means identification was DNA-assisted. Short notes are provided for some taxa. Photos of basidiocarps *in situ* are shown for some species (Figures 5–7).

All occurrence records considered in this study are attributed to 50 localities that are listed below and represented by numbers in brackets. Abbreviations used in these localities are as follows: Andijan Province—AP; Fergana Province—FP; Jizzakh Province; Karakalpakstan—K; Namangan Province—NMP; Navoi Province—NP; Qashqadaryo Province—QR; Samarkand Province—SP; Surkhandaryo Province—SRP; Syrdarya Province —SDR; Tashkent Province—TP; Tashkent botanical garden—TBG.

All territories of Uzbekistan except Kyzyl-kum desert (1); AP, Andijan District, Kutarma village (2); AP, Garden and Parks (3); AP, Shaxrixon district, Holdovonbek village (4); BP (5); FP (6); JP, Nurata State Reserve, Nurata Range, Pamir-Alay Mountain System (7); JP, Zaamin District, Zaamin National Park, Zaamin State Reserve in the South and South-east of the Turkestan Range (8); K, Lower-Amudarya Biosphere Reserve (9); Kyzyl-kum Desert (10); NMP, Chortoq District, Chortoq dam olishmaskani, Chortoq foothills (11); NMP, Haqiloobod District, Haquloobod village (12); NMP, Mingbuloq District, Qorasuv garden (13); NMP, National Parks and Gardens (14); NMP, Pop District, Chodaksay basin, Kurama Mountain Range of Western Tien Shan (15); NMP, Turaqurgon District, Kuymazor village, Pop and Chust foothills (16); NP, Sarmysh valley (17); NP. Tamdy District, Boymurot village, desert (18); QP, Hissar State Reserve in North-western of Hissar Range, Pamir-Alay Mountain System (19); QP, Yakkaobod village, Yakkabog forestry (20); SP, Zarafshan State Reserve, Zarafshan river valley, Pamir Mountains (21); SRP, Baysun District, Baysun village, Omonkhona, Baysun Mountain, South-western spurs of the Hissar Range in the Western part of the Pamir-Alay System (22); SRP, Baysun District, Darband village, Baysun Mountain, South-western spurs of the Hissar Range in the Western part of the Pamir-Alay System (23); SRP, Baysun District, Machay village, Baysun Mountain, South-western spurs of the Hissar Range in the Western part of the Pamir-Alay System (24); SRP, Hissar Range of Pamir-Alay Mountains (25); SRP, Surkhan State Reserve (26); SDR (27); TBG (28); Tashkent, olimlar shaxarchasi (29); TP, Angren, Yangibod village, South-eastern slope of Chatkal Mountain Range of Western Tien Shan (30); TP, Bekabad District, NW of Bekabad, Dalverzin village (31); TP, Bustonliq District, Beldersay, Greater Chimgan, Chatkal Mountain Range of Western Tien Shan (32); TP, Bustonliq District, Burchmulla village, Kulabsay, Western Tien Shan Mountains (33); TP, Bustonliq District, Gazalkent, spurs of the Western Tien Shan (34); TP, Bustonliq District, Kayinarsay and Sarvasay, Western Tien Shan (35); TP, Bustonliq District, Kuksu River, Pskem Mountain Range of Western Tien Shan (36); TP, Bustonliq District, Onaulgansoy, Pskem river, Pskem Mountain Range of Western Tien Shan (37); TP, Bustonliq District, Oqtosh village, Ugam Mountain Range of Western Tien Shan (38); TP, Bustonliq District, Xojikent village, Ugam Mountain Range of Western Tien Shan (39); TP, Bustonliq District, Xumson village, Xumsonsoy, Ugam Mountain Range of the Western Tien Shan (40); TP, Bustonliq District, Yubileyniy village, Chimyonsoy, Chimgan, Chatkal Mountain Range of Western Tien Shan (41); TP, Bustonliq District, Yusufhona village, Mazarsay, Charvak Reservoir, Western Tien Shan Mountains (42); TP, Karankulsay, Kungurbuka Mountain, Ugam Range of Western Tien Shan (43); TP, Oxangoron District, Oxangoron basin river (44); TP, Parkent District, Chatkal Biosphere Reserve, Chatkal Mountain Range of Western Tien Shan (45); TP, Parkent District, Kumyshkan village, Chatkal Mountain Range of Western Tien Shan Mountains (46); TP, Parkent District, Nivich and Qiziljar villages, Bashkyzylsay, Chatkal Biosphere Reserve, Chatkal Mountain Range of Western Tien Shan (47); TP, Tuyatashsoy, Western Tien Shan Mountains (48); TP, Ugam-Chatkal State Nature National Park, Western Tien Shan Mountains (49); TP, Yangikurgan village, Kurigansay river, Western Tien Shan Mountains (50).

**AGARICALES** Underw.

CYPHELLACEAE Lotsy

#***Chondrostereum purpureum*** (Pers.) Pouzar, Česká Mykol. 13(1): 17 (1959)

Specimen examined: (24): on *Acer pentapomicum* Stewart ex Brandis, 17 May 2016, YG-B01.

PTERULACEAE CORNER

#•***Radulomyces confluens*** (Fr.) M.P. Christ., Dansk bot. Ark. 19 (no. 2): 230 (1960)

Specimens examined: (39): on fallen rotten trunk, 2 Nov. 2011, YG006; (39): on trunks of angiosperm woody plant, 20 Nov. 2013, YGcor-80; (28): on dead stump, 2 Sept. 2013, YG-G43.

NIACEAE Jülich

^∗^***Merismodes anomala*** (Pers.) Singer, Agaric. mod. Tax., Edn 3 (Vaduz): 665 (1975)

Specimen examined: (49): on dead branch of *Prunus* sp., 30 Apr. 1988, A. Kollom, TAAM127589.

*Note*: In TAAM, this specimen was originally labeled as *Cyphellopsis anomala* (Pers.) Donk.

SCHIZOPHYLLACEAE Quél.

•***Schizophyllum commune*** Fr., Observ. mycol. (Havniae) 1: 103 (1815)

Specimens examined: (40): on *Morus alba* L., 26 May 2011, YG047; (37): on dead trunk of *Juglans regia* L., 20 Sept. 2014, YG/PS169; (45): on a dry branch of *Celtis australis* subsp. *caucasica* (Willd.) C.C. Towns., 30 Apr. 1980, A. Kollom, TAAM127588; (20): on *Populus* sp., Sept. 2012, YG-J2.

Literature: Khalikova (1989), (49): on *Salix pentandra* L. and *Prunus armeniaca* L.; Iminova (2009), (4): on *Salix pentandra* L., *Ìàlus domestica, Prunus armeniaca*, Jun. 2000, Jul. 2004.

**ATHELIALES** Jülich

ATHELIACEAE Jülich

#•***Athelia arachnoidea*** (Berk.) Jülich, Willdenowia, Beih. 7: 53 (1972)

Specimens examined: (38): on fallen angiosperm branch, 3 Sept. 2013, YG-G23; (38): YG-G44; (37): on living branches of *Crataegus* sp., 18 Jun. 2014, YG/PS154; (8): on dried branches of *Lonicera paradoxa* Pojark., 26 May 2018, YG1111.

**CANTHARELLALES** Gäum.

HYDNACEAE Chevall.

#***Sistotrema coroniferum*** (Höhn. and Litsch.) Donk, Fungus, Wageningen 26: 4 (1956)

Specimen examined: (37): on branches of *Betula* tree, 19 Jun. 2014, YG/PS95.

**CORTICIALES** K.H. Larss.

CORTICIACEAE Herter

#***Corticium roseum*** Pers., Neues Mag. Bot. 1: 111 (1794)

Specimen examined: (35): on stumps of *Juglans regia*, 31 Aug. 1963, A. Raitviir, TAAM043491.

¤•***Vuilleminia*** sp. Parmasto, Eesti NSV Tead. Akad. Toim., Biol. seer 16(4): 391 (1967)

Specimen examined: (36): on bark and at base of a trunk of *Lonicera* sp., E. Parmasto, 25 Apr. 1982, TAAM104410.

**GLOEOPHYLLALES** Thorn

GLOEOPHYLLACEAE Jülich

•***Gloeophyllum abietinum*** (Bull.) P. Karst., Bidr. Känn. Finl. Nat. Folk 37: 80 (1882)

Specimens examined: (49): on trunk of *Juniperus* sp., 23 Apr. 1982, A. Kollom, TAAM127397.

Literature: Baltaeva (1992, 1993), [(45): on *Pinus* sp., 10 May 1987; (8): on *Pinus* sp., 17 Jun. 1988; (7): on dried stem of *Pinus* sp., 28 Jul. 1988; (21): on stem of *Picea* sp., 21 Oct. 1988; (19): on *Juniperus* sp., 29 Jul. 1989].

*Note*: Although the species is easily recognizable in the field, to our surprise, we failed to find it.

***Gloeophyllum odoratum*** (Wulfen) Imazeki, Bull. Tokyo Sci. Mus. 6: 75 (1943)

Literature: Baltaeva (1992), [(19): on wet trunk of *Biota* sp., 5 Sept. 1990].

***Gloeophyllum trabeum*** (Pers.) Murrill, N. Amer. Fl. (New York) 9(2): 129 (1908)

Literature: Baltaeva (1992, 1993), [(45): on stump and trunk of *Quercus* sp., 1 Jun. 1987; (8): on strum of unknown woody plants, 1 Jun. 1987; (7): on trunk of deciduous tree, 4 Oct. 1988; (21): on fallen stem of angiosperm, 22 Jun. 1988; (19): on dried stem of *Quercus* sp., 19 Mar. 1988; (9): on *Quercus* sp., 7 May 1988].

**POLYPORALES** Gäum.

MERULIACEAE Rea

***Abortiporus biennis*** (Bull.) Singer, Mycologia 36(1): 68 (1944)

Literature: Baltaeva (1992), [(45): on dead attached branches of living *Quercus* sp., 12 Sept. 1990].

•***Aurantiporus fissilis*** (Berk. and M.A. Curtis) H. Jahn ex Ryvarden, Polyp. N. Eur. (Oslo) 2: 222 (1978).

≡ *Tyromyces fissilis* (Berk. and M.A. Curtis) Donk.

Specimen examined: (28): on living stem of *Juglans regia*, 9 Jun. 2014, YG/bot3.

Literature: Kravtzev (1950), (as *Tyromyces fissilis*, (19): on stem of *Malus* sp.); Baltaeva (1992, 1993), (as *Tyromyces fissilis*, (1): on various deciduous wood).

•***Bjerkandera adusta*** (Willd.) P. Karst., Meddn Soc. Fauna Flora fenn. 5: 38 (1879)

Specimens examined: (40): on dead stump of *Juglans regia*, 26 May 2011, YG012; (37): on dried *Juglans regia* log, 20 Jun. 2014, YG/PS172; (37): on dried trunk of angiosperm wood, 20 Jun. 2014, YG/PS183; (29): on dead stump of *Prunus armeniaca*, 2 Apr. 2013, YG-G41; (28): on unknown wood, 3 Sept. 2013, YG/bot23a; (47): on trunk of *Populus alba* L., 4 May 1988, I. Parmasto, TAAM126292; (19): on fallen trunk of *Populus* sp., 15 Jun. 2013, YG-O1.

Literature: Panfilova and Gaponenko (1963), [(44): on dried log of *Salix* sp.], Khalikova (1989), [(34): on decaying *Prunus armeniaca* log], Baltaeva (1992, 1993), [(49): on trunk of *Salix* sp., 13 Aug. 1986; (45): on dried stem and trunk of *Populus* sp., 25 Jul. 1986; (19): on decaying *Quercus* log, 6 May 1986; (6): on *Tilia* sp., 29 May 1989; (19): on stump of *Ulmus* sp., 28 Jun. 1988].

*Note*: This species is very common and occurs on dead and senescent deciduous wood. We mostly found it on *Juglans, Populus*, and *Prunus* species in Uzbekistan.

***Bjerkandera fumosa*** (Pers.) P. Karst., Meddn Soc. Fauna Flora fenn. 5: 38 (1879)

Literature: Baymuratova (1963), [(27): on trunk of *Quercus* sp., 1962]; Khalikova (1989), [(28): on *Quercus* sp., Jun. 1983; (49): on dried trunk of *Populus* sp., Jul. 1984]; Baltaeva (1992, 1993), [(49): on *Populus* sp., 19 May 1985; (45): on stem of *Populus* sp., 6 Jun. 1986; (1): on trunk of hardwood].

***Gelatoporia dichroa*** (Fr.) Ginns, Index Fungorum 156: 1 (2014)

≡ *Gloeoporus dichrous* (Fr.) Bres.

Literature: Panfilova and Gaponenko (1963), (as *Gloeoporus dichrous*, (49): dried stem of *Morus alba*, 1962); Baltaeva (1992, 1993), (as *Gloeoporus dichrous*, (45): on decaying *Picea* sp., 4 Jul. 1988; (8): on dried stem of *Pinus* sp., 6 Aug. 1987; (19): on *Pinus* sp., 18 Jul. 1989; (26): on *Pinus* sp., 10 Aug. 1989).

¤•***Hyphoderma* sp.**

Specimen examined: (37): on decaying branch of *Betula* sp., 19 Jun. 2014, YG/PS133.

***Irpex lacteus*** (Fr.) Fr., Elench. fung. (Greifswald) 1: 142 (1828)

Literature: Kleyner (1958), [(25): on branch of *Pyrus* sp., 1957]; Baltaeva (1992, 1993), [(19): on stump of *Populus* sp., 19 Aug. 1985, (19): on *Ulmus* sp., 12 Aug. 1989; (8): on branch of *Alnus* sp., 9 Sept. 1986; (7): on *Quercus* sp., 4 Aug. 1987; (45): on dried stem of *Salix* sp., 16 Oct. 1988].

***Irpiciporus litschaueri*** (Lohwag) Zmitr., Folia Cryptogamica Petropolitana (Sankt-Peterburg) 6: 105 (2018).

≡ *Spongipellis litschaueri* Lohwag

Literature: Baltaeva (1992, 1993), (as *Spongipellis litschaueri*, 7: on stem of *Malus* sp., 7 Aug. 198; (7): on trunk of *Quercus* sp., 14 Aug. 1986; (8): on *Fraxinus* sp., 29 Jul. 1988; (9): on *Quercus* sp., 19 Aug. 1987; (45): on stems of *Ulmus* sp., 4 Sept. 1987; (21): on angiosperm fallen wood, 27 Sept. 1987; (26): on dead *Fraxinus* branch, 3 Sept. 1989).

^∗^***Mycoacia aurea*** (Fr.) J. Erikss. and Ryvarden, Cortic. N. Eur. (Oslo) 4: 877 (1976).

≡ *Phlebia aurea* (Fr.) Nakasone

Specimen examined: (28): on a fallen rotten deciduous trunk, 20 Apr. 1982, E. Parmasto, TAAM104260.

*Note*: In TAAM, this specimen was originally labeled as *Phlebia aurea*.

•***Phlebia bresadolae*** Parmasto, Eesti NSV Tead. Akad. Toim., Biol. seer 16(4): 390 (1967)

Specimens examined: (37): on fallen branch of angiosperm, 18 Jun. 2014, YG/PS189; (37): on unknown woody plants branch, 19 Jun. 2014, YG/PS89.

¤•***Phlebia* sp.** (P. Karst.) Ryvarden, Rept. Kevo subarct. Res. Stn 8: 151 (1971)

Specimens examined: (39): on dead hardwood, 2 Nov. 2011, YG326; (40): on living *Crataegus pseudoheterophylla* subsp. *turkestanica*, 26 May 2011, YG64.

^∗^•***Phlebia rufa*** (Pers.) M.P. Christ., Dansk bot. Ark. 19(no. 2): 164 (1960)

Specimen examined: (40): on living stems of *Robinia pseudoacacia* L., 26 May 2011, YG77.

***Resiniporus resinascens*** (Romell) Zmitr., Folia Cryptogamica Petropolitana (Sankt-Peterburg) 6: 98 (2018)

≡ *Ceriporiopsis resinascens* (Romell) Domański

Literature: Baltaeva (1992, 1993), (as *Ceriporiopsis resinascens* (Romell) Domański, (45): on fallen branch of *Betula* sp., 7 May 1987; (19): on fallen log of *Populus* sp., 30 May 1987).

***Sarcodontia spumea*** (Sowerby) Spirin, Mycena 1(1): 64–71 (2001)

≡ *Spongipellis spumeus* (Sowerby) Pat.

Literature: Baltaeva (1992, 1993), (as *Spongipellis spumeus* (Sowerby) Pat., (45): on fallen stem of *Ulmus* sp., 4 Sept. 1987; (21): on trunk of *Ulmus* sp., 27 Sept. 1987; (7): on stem of *Malus* sp., 7 Aug. 1986, (7): on *Quercus* sp., 19 Aug. 1989; (8): on *Fraxinus* sp., 29 Jul. 1988; (19): on dried trunk of *Fraxinus* sp., 3 Sept. 1989; (9): on dried strum of *Quercus* sp., 14 Aug. 1986).

^∗^***Steccherinum ciliolatum*** (Berk. and M.A. Curtis) Gilb. and Budington, J. Ariz. Acad. Sci. 6(2): 97 (1970).

Literature: Gafforov et al. (2017), [(45): on dead trunk of *Prunus spinosissima* (Bunge) Franch., 29 Apr. 1988, A. Kollom, TAAM127581].

FOMITOPSIDACEAE Jülich

***Amyloporia sinuosa*** (Fr.) Rajchenb., Gorjón and Pildain, Aust. Syst. Bot. 24(2): 117 (2011)

≡ *Antrodia sinuosa* (Fr.) P. Karst.

Literature: Baltaeva (1992), (as *Antrodia sinuosa* (Fr.) P. Karst., (21): on wet trunk of *Pinus* sp., 15 Aug. 1990).

***Antrodia albida*** (Fr.) Donk, Persoonia 4(3): 339 (1966)

Literature: Kravtzev (1950), [(27): on *Quercus* sp.]; Baltaeva (1992, 1993), [(49): on bark of *Quercus* fallen branches, 5 Apr. 1987; (45): on fallen *Betula* trunk, 18 May 1986; (26): on stumps of *Populus* sp., 25 Jun. 1988; (8): on wet woody plant, 5 Apr. 1987].

***Antrodia heteromorpha*** (Fr.) Donk, Persoonia 4(3): 339 (1966)

Literature: Baltaeva (1992), [(21): on rotten *Pinus* fallen trunk, 17 Aug. 1990].

***Antrodia xantha*** (Fr.) Ryvarden, Norw. Jl Bot. (20): 8 (1973)

Specimens examined: (33): on trunk of *Juniperus polycarpos* var. *seravschanica*, 22 Apr. 1982, E. Parmasto, TAAM104400; (50): on rotten trunk of *Juniperus semiglobosa* Regel, 24 Apr. 1982, E. Parmasto, TAAM104301; (33): on fallen rotten trunk of *Juniperus semiglobosa*, 24 Apr. 1982, E. Parmasto, TAAM104289.

Literature: Baltaeva (1992, 1993), [(45): on *Pinus* sp., 19 Aug. 1988; (19): on *Juniperus polycarpos* var. *seravschanica*, 15 Aug. 1988; (21): on *Pinus* sp., 3 Jul. 1989; (7): on stumps of *Picea* sp., 13 Jul. 1989; (7): on *Juniperus* sp., 15 Jul. 1989; (49): on *Pinus* sp., 20 Jul. 1989].

*Note*: This species appears to be common in the study area. However, we did not find fresh specimens during our field trips.

#***Brunneoporus juniperinus*** (Murrill) Zmitr., Folia Cryptogamica Petropolitana (Sankt-Peterburg) 6: 86 (2018).

Specimen examined: (36): on base of tree of *Juniperus semiglobosa*, 25 Apr. 1982, M. Khalikova, TAAM104433.

*Note*: In TAAM, this specimen was originally labeled as *Antrodia juniperina* (Murrill) Niemelä and Ryvarden.

***Climacocystis borealis*** (Fr.) Kotl. and Pouzar, Česká Mykol. 12(2): 103 (1958)

Literature: Baltaeva (1992, 1993), [(45): on old decaying trunks of *Pinus* sp., 24 Jul. 1988; (26): on fallen trunk of *Picea* sp., 26 Aug. 1989].

***Daedalea quercina*** (L.) Pers., Syn. meth. fung. (Göttingen) 2: 500 (1801)

Literature: Baltaeva (1992), [(21): on stem of *Juglans regia*, 15 Aug. 1990].

^∗^•***Flavidoporia pulverulenta*** (B. Rivoire) Audet, Mushrooms nomenclatural novelties 4: [1] (2017)

Specimens examined: (37): on trunk of *Salix* sp., 19 Jun. 2014, YG/PS167; (37): on rotten of branch of *Salix alba* L., 11 Sept. 2016, YG1110.

***Fomitopsis betulina*** (Bull.) B.K. Cui, M.L. Han and Y.C. Dai, in Han, Chen, Shen, Song, Vlasák, Dai and Cui, Fungal Diversity 80: 359 (2016)

≡ *Piptoporus betulinus* (Bull.) P. Karst.

Literature: Panfilova and Gaponenko (1963); Khalikova (1989), (as *Piptoporus betulinus* (Bull.) P. Karst., (49): on stem of *Betula* sp.); Baltaeva (1992, 1993), (as *Piptoporus betulinus*, (45): on trunk of *Betula* sp., 14 Jul. 1987; (19): on wet *Betula* stems, 5 Aug. 1987; (26): on *Betula* sp., 30 Jun. 1988; (8): on dried stems of *Betula* sp., 24 Aug. 1989); Iminova (2009), (as *Piptoporus betulinus*, (3): on *Betula tianschanica*, Jul.–Nov. 2003–2005).

***Fomitopsis pinicola*** (Sw.) P. Karst., Meddn Soc. Fauna Flora fenn. 6: 9 (1881)

Specimen examined: (48): on *Juniperus polycarpos* var. *seravschanica*, J.K. Rotkevich, Jul. 1956, TASM002.

Literature: Baltaeva (1992, 1993), [(45): on living trunk of *Pinus* sp., 25 Jul. 1986; (7): on living trunk of *Pinus* sp., 11 Jun. 1987; (8): on living stem of *Picea* sp.; (21): on living *Pinus* tree, 3 Aug. 1987; (19): on fallen stem of conifer tree, 19 Jul. 1988].

***Laetiporus sulphureus*** (Bull.) Murrill, Annls mycol. 18(1/3): 51 (1920)

Specimens examined: (28): on stem of *Salix* sp., 2 Sept. 2011, YG031; (28): on dried strum angiosperm wood, 3 Oct. 2011, YG041; (24): on *Acer tataricum* subsp. *semenovii* (Regel and Herder) A.E. Murray, 20 Aug. 2016, YG-B10.

Literature: Panfilova and Gaponenko (1963); Khalikova (1989), [(49): on living stem of *Prunus mahaleb* L. and *Juglans regia*]; Baltaeva (1992, 1993), [(45): on trunk of *Robinia pseudoacacia* L., 16 Oct. 1986; (1): on trunks of *Acacia*, 30 Jul. 1989; (1): on trunk of *Quercus*, 12 Sept. 1989].

***Neoantrodia serialis*** (Fr.) Audet, Mushrooms nomenclatural novelties 6: [2] (2017)

≡ *Antrodia serialis* (Fr.) Donk

Literature: Baltaeva (1992), (as *Antrodia serialis*, (19): on dried of *Pinus* trunk, 6 Apr. 1989).

***Phaeodaedalea incerta*** (Curr.) Tura, Zmitr., Wasser and Spirin, Biodiversity of the Heterobasidiomycetes and non-gilled Hymenomycetes (former Aphyllophorales) of Israel: 401 (2011).

= *Gloeophyllum sprucei* (Berk.) Teixeira

Literature: Baltaeva (1992), (as *Gloeophyllum sprucei*, (19): on wet branches of *Pinus* sp., 25 Jul. 1990).

***Phaeolus schweinitzii*** (Fr.) Pat., Essai Tax. Hyménomyc. (Lons-le-Saunier): 86 (1900)

Literature: Baltaeva (1992, 1993), [(49): on dried fallen stem of *Pinus* sp., 30 Jul. 1989; (45): on trunk of *Picea* sp., 29 Jul. 1989]; Iminova (2009), [(3): on *Platanus orientalis*, Jun.–Jul. 2005].

^∗^•***Pilatoporus ibericus*** (Melo and Ryvarden) Kotl. and Pouzar, Cryptog. Mycol. 14(3): 217 (1993)

Specimens examined: (49): on trunk of angiosperm tree, 3 Sept. 2013, YG-G24.

***Postia caesia*** (Schrad.) P. Karst., Revue mycol., Toulouse 3(no. 9): 19 (1881)

≡ *Oligoporus caesius* (Schrad.) Gilb. and Ryvarden

Literature: Baltaeva (1992, 1993), (as *Oligoporus caesius*, (45): on stump of *Picea* sp., 17 Oct. 1987, 6 Nov. 1987; (8): on trunk of *Pinus* sp., 28 Oct. 1987; (7): on *Pinus* fallen branch, 12 Nov. 1988).

***Postia sericeomollis*** (Romell) Jülich, Persoonia 11(4): 423 (1982)

≡ *Oligoporus sericeomollis* (Romell) Bondartseva

= *Chaetoporellus litschaueri* (Pilát) Bondartsev

Literature: Baltaeva (1992, 1993), (as *Oligoporus sericeomollis, Chaetoporellus litschaueri*), [(45): on stump of *Pinus* sp., 18 Sept. 1986; (19): on *Pinus* sp., 21 Jun. 1989; (8): on trunk of *Picea* sp., 9 May 1987; (7): on fallen branch of *Picea* sp., 9 May 1987].

***Postia stiptica*** (Pers.) Jülich, Persoonia 11(4): 424 (1982)

≡ *Oligoporus stipticus* (Pers.) Gilb. and Ryvarden

Literature: Baltaeva (1992, 1993), (as *Oligoporus stipticus*, (45): on stump of *Pinus* sp, 9 Oct. 1987; (19): on *Pinus* sp., 24 Oct. 1987; (21): on fallen trunk of *Picea* sp., 6 Nov. 1988).

***Osteina obducta*** (Berk.) Donk, Schweiz. Z. Pilzk. 44: 86 (1966)

≡ *Oligoporus obductus* (Berk.) Gilb. and Ryvarden

Literature: Baltaeva (1992), (as *Oligoporus obductus*, (21): on root of *Pinus* sp., 15 Aug. 1990).

***Rhodofomes roseus*** (Alb. and Schwein.) Vlasák, Ćeská Mykol. 44(4): 235 (1990)

≡ *Fomitopsis rosea* (Alb. and Schwein.) P. Karst

Specimens examined: (45): on conifer fallen trunk, 15 Jun. 1980, S.S. Ramazanova, N4 (TASM).

Literature: Baltaeva (1992, 1993), (as *Fomitopsis rosea*, (45): on dead standing *Picea* trunk, 7 Apr. 1987; (45): on *Picea* sp., 21 May 1988; (8): on trunk of *Picea* sp., 9 Jun. 1987; (7): on fallen branch of *Pinus* sp., 30 May 1988; (14): on *Pinus* sp., 5 May 1989).

•***Subantrodia uzbekistanica*** (Yuan, Gafforov and F. Wu) Audet, Mushrooms nomenclatural novelties 9: [1] (2017).

≡ ***Antrodia uzbekistanica*** Yuan, Gafforov and F. Wu

Specimens examined: (8): on Juniper tree rotten wood, 8 Sept. 2016, YG1014; (8): on trunk of *Juniperus* sp., 4 Sept. 2017, YG1103; (8): on rotten stem of *Juniperus polycarpos* var. *seravschanica* (Kom.) Kitam., 10 Sept. 2017, YG1107; (8): on unknown woody branches, 9 Sept. 2016, YG1100.

Literature: Yuan et al. (2017), (same place: as *Antrodia uzbekistanica*).

PHANEROCHAETACEAE Jülich

^∗^•***Byssomerulius corium*** (Pers.) Parmasto, Eesti NSV Tead. Akad. Toim., Biol. seer 16(4): 383 (1967)

Specimens examined: (28): on angiosperm fallen branch, 2 Sept. 2013, YG-G21; (7): on dried branch of *Prunus vulgaris* L., 28 Aug. 2013, YG-X3.

^∗^***Ceriporia purpurea*** (Fr.) Donk, Proc. K. Ned. Akad. Wet., Ser. C, Biol. Med. Sci. 74(1): 28 (1971)

Specimen examined: (45): on a deciduous tree, 1 May 1988, A. Kollom, TAAM127605.

•***Ceriporiopsis gilvescens*** (Bres.) Domański, Acta Soc. Bot. Pol. 32(4): 731 (1963)

≡ *Tyromyces gilvescens* (Bres.) Ryvarden

Specimens examined: (28): on dried angiosperm wood, 14 Oct. 2011, YG046; (36): on base of rotten trunk of wood, 8 Jun. 2011, YG049; (32): on *Juniperus pseudosabina* Fisch. et C.A. Mey., 26 May 2011, YG008.

Literature: Baltaeva (1992, 1993), (as *Tyromyces gilvescens*, (45): on fallen branch of *Populus* sp., 2 May 1987; (19): on *Quercus* trunk, 19 Aug., 1988; (26): on died fallen of *Populus* sp., 1 Sept. 1988; (9): on rotten trunk of *Populus* sp., 10 Jul. 1987; (21): on rotten trunk of *Malus* sp., 21 Jul. 1986; (6): on dead stump and trunk of deciduous wood, 13 Apr. 1986).

***Ceriporiopsis mucida*** (Pers.) Gilb. and Ryvarden, Mycotaxon 22(2): 364 (1985)

Literature: Baltaeva (1992), [(5): on dried branch of *Populus* sp., 28 Aug. 1990].

#***Efibula tuberculata*** (P. Karst.) Zmitr. and Spirin, in Zmitrovich, Malysheva and Spirin, Mycena 6: 33 (2006)

Specimen examined: (18): on fallen trunk of *Haloxyllon* sp., 6 Apr. 1979, K. Kalamees, TAAM120642.

*Note*: In TAAM, this specimen was originally labeled as *Athelia* sp.

POLYPORACEAE Fr. ex Corda

***Cerioporus mollis*** (Sommerf.) Zmitr. and Kovalenko, Int. J. Med. Mushrooms 18(1): 33 (2016)

≡ *Datronia mollis* (Sommerf.) Donk

Literature: Baltaeva (1992, 1993), (as *Datronia mollis*, (45): on wet dead trunk of *Populus* sp., 10 May 1985; (19): on *Populus* sp., 21 Apr. 1986; (26): on *Populus* sp., 17 Jun. 1987).

***Cerioporus squamosus*** (Huds.) Quél., Enchir. fung. (Paris): 167 (1886)

≡ *Polyporus squamosus* (Huds.) Fr.

Specimens examined: (50): on trunk of *Juglans regia*, 24 Apr. 1982, A. Kollom, TAAM127413; (36): on dried trunk and stem of angiosperm woody plant, 6 Jun. 2011, YG026; (37): on rotten trunk of *Acer tataricum* subsp. *semenovii*, 2 Sept. 2017, YG20170902; (22): on dried stump of *Populus alba*, 13 May 2015, YG-B02; (23): on *Acer* sp., 17 May 2015, YG-B05.

Literature: Panfilova and Gaponenko (1963), Akhmedova, 1966 (as *Polyporus squamosus*, (49): on trunk of *Juglans regia*); Khalikova (1989), (as *Polyporus squamosus*, (49): on *Pistacia* sp.); Baltaeva (1992, 1993), (as *Polyporus squamosus*, (1): on stump collar of *Quercus*, sp., 1988, on fallen big branch of *Juglans regia*, 1989); Iminova (2009), (as *Polyporus squamosus*, (3): on various woody plants, May–Jun. 2000–2004).

*Note*: This species is widespread on angiosperm woody plants across study area.

***Cerioporus varius*** (Pers.) Zmitr. and Kovalenko, Int. J. Med. Mushrooms 18(1): 33 (2016)

≡ *Polyporus varius* (Pers.) Fr.

Literature: Baltaeva (1992), (as *Polyporus varius*, (45): on deadwood stem of *Quercus* sp., 23 Jun. 1990; (19): on fallen stem of *Lonicera* sp., 10 Aug. 1990).

•***Cerrena unicolor*** (Bull.) Murrill, J. Mycol. 9(2): 91 (1903)

Specimens examined: (28): on dried fallen stem of angiosperm tree, 2 Sept. 2013, YG-G28; (32): on *Acer tataricum* subsp. *semenovii*, 15 May 2011, YG18; (32): on dried stem of *Acer tataricum* subsp. *semenovii*, 15 May 2011, YG027; (38): on *Crataegus pseudoheterophylla* subsp. *turkestanica*, 1 Jun. 2011, YG003; (41): on a trunk of *Juglans regia*, 22 Apr. 1982, E. Parmasto, TAAM104271; (41): on dry twig of *Acer* sp., 22 Apr. 1982, A. Kollom, TAAM127385; (50): on dead trunk of *Salix* sp., 24 Apr. 1982, A. Kollom, TAAM127405; (37): on *Acer tataricum* subsp. *semenovii*, 19 Sept. 2014, YG/PS79; (45): on dry branch of *Celtis australis* subsp. *caucasica*, 29 Apr. 1988, A. Kollom, TAAM127582; (45): on *Celtis australis* subsp. *caucasica*, 3 May 1988, A. Kollom, TAAM127632; (45): on dry trunk of *Celtis australis* subsp. *caucasica*, 1 May 1988, I. Parmasto, TAAM126263; (47): on trunk of *Prunus mahaleb*, 29 Apr. 1988, I. Parmasto, TAAM126248.

Literature: Akhmedova (1966), [(49): on *Populus* sp.]; Khalikova (1989), [(49): on trunk of *Populus* sp., Jul. 1988]; Baltaeva (1992, 1993), [(1): on stump of *Quercus* sp., *Populus* sp., *Salix* sp., Jul.–Aug. 1988–1989].

*Note*: This species is widespread and causes damage to *Acer tataricum* subsp. *semenovii* trees in Tien Shan Mountain.

***Coriolopsis gallica*** (Fr.) Ryvarden, Norw. Jl Bot. 19: 230 (1973)

≡ *Funalia gallica* (Fr.) Bondartsev and Singer

Literature: Panfilova and Gaponenko (1963), (as *Funalia gallica*, (49): on *Quercus* sp.); Khalikova (1989), (as *Funalia gallica*, (28): on stumps and dried trunks of *Fraxinus americana* L., Jun. 1986, Sept. 1986, Dendropark, May, 1987); Baltaeva (1992, 1993), [(45): on dried stem and branches of *Quercus* sp., 6 Jun. 1985, (45): on *Quercus* sp., 3 Jun. 1988, (45): on *Salix* sp., 17 Jul. 1987, (47): on *Populus* sp., 27 Jul. 1987; (8): on *Fraxinus* sp., 4 Jul. 1989; (7): on *Fraxinus* sp., 1 Aug. 1989; (19): on stem of *Fraxinus* sp., 20 Jul. 1986; (21): on trunk of *Populus* sp., 27 Jul. 1987; (6): on *Quercus* sp., 3 Jun. 1988; (9): on *Populus tremula* L., 1 Aug. 1989]; Iminova (2009), (as *Funalia gallica*, (3): on fallen trunks of *Platanus orientalis* L., May 2005, Sept. 2005).

***Daedaleopsis confragosa*** (Bolton) J. Schröt., in Cohn, Krypt.-Fl. Schlesien (Breslau) 3.1(25–32): 492 (1888) [1889]

Literature: Baltaeva (1992), [(45): on decaying stem of *Betula* sp., 11 Aug. 1990; (6): on *Betula* sp., 12 Sept. 1991; on fallen tree of *Betula* sp., 6 Aug. 1990, on *Betula* sp., 19 Jul. 1988].

***Dichomitus squalens*** (P. Karst.) D.A. Reid, Revta Biol., Lisb. 5(1–2): 150 (1965) [1964–5]

Literature: Baltaeva (1992, 1993), [(45): on the bark of *Pinus* sp., 9 May 1987; (21): on *Biota* sp., 21 Jun. 1989; (21): on *Pinus* sp., 14 May 1988; (7): on *Pinus* sp., 9 Jun. 1989; (8): on *Picea* sp., 20 Jun. 1988; (19): on living stem of old *Biota* sp., 19 Jul. 1988].

***Diplomitoporus flavescens*** (Bres.) Domański, Acta Soc. Bot. Pol. (39): 191 (1970)

≡ *Antrodia flavescens* (Bres.) Ryvarden

Literature: Khalikova (1989), (as *Antrodia flavescens*, (49): on fallen logs of *Picea* sp., 22 Apr. 1980); Baltaeva (1992), (as *Diplomitoporus flavescens*), Baltaeva, 1993 (as *Antrodia flavescens*, (7): on *Picea* stump, 20 Jul. 1987; (26): on *Juniper* fallen stems, 17 Jun. 1987; (21): on *Pinus* trunk, 21 Jun. 1989).

***Fibroporia vaillantii*** (DC.) Parmasto, Consp. System. Corticiac. (Tartu): 177 (1968)

≡ *Antrodia vaillantii* (DC.) Ryvarden

Literature: Baltaeva (1992, 1993), (as *Antrodia vaillantii*, (45): on trunk of *Picea* sp., 18 Aug. 1989).

•***Fomes fomentarius*** (L.) Fr., Summa veg. Scand., Sectio Post. (Stockholm): 321 (1849)

Specimens examined: (28): on living stem of *Populus* sp., 3 Sept. 2013, YG/bot2; (28): on decaying trunk of identified angiosperm, 4 Sept. 2013, YG/bot4; (40): on living trunk of *Juglans regia*, 26 May 2011, YG014; (28): on unknown wood, 7 Nov. 2014, YG-60, *ibit*., on unknown trunk decaying wood, YG-70; (38): on *Juglans regia*, 13 Sept. 2012, YG/Un2; (37): on dried *Juglans regia* trunk, 14 Sept. 2014, YG/PS174; (23): on living stem of *Juglans regia*, 13 Aug. 2015, YG-B03; (22): on dried stem of *Salix alba*, 17 Aug. 2016, YG-B04.

Literature: Panfilova and Gaponenko (1963); Akhmedova (1966); Khalikova (1989), [(49): on stem and trunk decaying and living *Juglans regia*]; Baltaeva (1992), [(1): on dead and living deciduous trees on *Malus* sp., *Quercus* sp., *Populus* sp.]; Iminova (2009), [(3): on living stem of *Salix wilhelmsiana* M. Bieb., Sept. 2000; (4): on died trunk of *Salix alba*, Oct. 2001].

*Note*: This species is widespread on living trees in the study area.

***Hapalopilus rutilans*** (Pers.) Murrill, Bull. Torrey bot. Club 31(8): 416 (1904)

= *Hapalopilus nidulans* (Fr.) P. Karst.

Literature: Baltaeva (1992, 1993), (as *Hapalopilus nidulans*, (49): on dead branch of *Betula* sp., 1 Jul. 1989, (49): on fallen stem of *Populus* sp., 14 Jul. 1989; (19): on fallen strums of *Populus* sp., 29 Jul. 1989).

***Lentinus arcularius*** (Batsch) Zmitr., Int. J. Med. Mushrooms 12(1): 88 (2010)

≡ *Polyporus arcularius* (Batsch) Fr.

Literature: Khalikova (1989), (as *Polyporus arcularius*, (45): on dead branch of *Juglans regia*, May, 1982, Nov. 1983; (41): on *Salix interior* Rowlee, Apr.–May 1983); Iminova (2009), (as *Polyporus arcularius*; (11): dried trunk of *Juglans regia*, Apr. 2000, Nov. 2004).

***Lentinus brumalis*** (Pers.) Zmitr., Int. J. Med. Mushrooms 12(1): 88 (2010)

≡ *Polyporus brumalis* (Pers.) Fr.

Literature: Schwartzman (1964), (as *Polyporus brumalis*, (49): on *Celtis australis* subsp. *Caucasica*); Baltaeva (1993), (as *Polyporus brumalis*, (21): on stem of branch of *Salix* sp., 21 Jul. 1987; (3): on *Salix* sp., 15 Aug. 1987; (45): on *Betula* sp., 10 May 1987; (19): on *Betula* sp., 20 Jul. 1988; (8): on *Populus* sp., 6 Sept. 1988; (7): on small branches of *Salix* sp., 24 Aug. 1987; (27): 20 Jul. 1988; (10): on dried stem of *Populus* sp., 24 Aug. 1989).

***Lentinus substrictus*** (Bolton) Zmitr. and Kovalenko, Int. J. Med. Mushrooms 18(1): 35 (2016)

= *Polyporus ciliatus* Fr.

Literature: Baltaeva (1992), (as *Polyporus ciliatus*, (45): on branches of *Salix* sp., 15 Aug. 1987; (8): on *Salix* sp., 21 Aug. 1987; (7): on strum and branch of *Populus* sp., 6 Nov. 1988; (19): on *Betula* fallen trumps, 10 May 1987; (21): on *Betula* sp., 30 Aug. 1988; (3): on *Populus* sp., 24 Aug. 1989).

•***Lentinus tigrinus*** (Bull.) Fr., Syst. orb. veg. (Lundae) 1: 78 (1825)

≡ *Panus tigrinus* (Fr.) Sing.

Specimens examined: (28): on *Salix* sp., 24 Apr. 1989, K. Kalamees, TAAM144150; (28): on stump of *Lonicera* sp., 20 Apr. 1982, A. Kollom, TAAM104259; (40): on decaying *Juglans regia*, 26 May YG029; (33): on fallen trunk of angiosperm, 23 Apr. 1982, M. Khalikova, TAAM104290; (33): on trunk of *Juglans regia*, 23 Apr. 1982, E. Parmasto, TAAM104406; (33): on strum of *Salix* sp., 23 Apr. 1982, A. Kollom, TAAM127396; (41): on stump of unidentified wood, 23 Apr. 1982, A. Kollom, TAAM127381; (41): on trunk of angiosperm tree, 22 Apr. 1982, M. Khalikova, TAAM104275; (47): on *Salix* sp., 1 May 1988, A. Kollom, TAAM127603; (37): on trunk of *Malus domestica*, 18 Jun. 2014, YG/PS162; (17): on *Salix* sp., 7 May 1976, TAAM094856; (17): on stump of *Prunus armeniaca*, 7 May 1976, K. Kalamees and others, TAAM094857; (17): on *Acer* tree trunk, 7 May 1976, K. Kalamees and others, TAAM094847; (20): on dried trunks of angiosperm wood, 13 Jun. 2013, YG-J7.

Literature: Panfilova and Gaponenko (1963), [(44): on living trunk and stems of *Lonicera* sp., and on fallen dried trunk of *Acer* sp.]; Khalikova (1989), [(34): on dried stem of *Malus domestica*]; Iminova (2009), [(3): on *Salix wilhelmsiana*, on *Populus euphratica* Oliv., on living stem of *Populus talassica* Kom., Nov. 2005]; Iminova (2009), (as *Panus tigrinus*, (4): on *Salix linearifalia* Wolf.).

*Note*: This is a widespread species in the study area.

***Lenzites betulinus*** (L.) Fr., Epicr. syst. mycol. (Upsaliae): 405 (1838) [1836–1838]

Literature: Baltaeva (1992, 1993), [(45): on fallen twigs of *Betula* sp., 17 Aug. 1987; (26): on *Populus* sp., 22 Jul. 1988; (10): on *Populus* sp., 10 Sept. 1988; (21): on *Salix* sp., 26 Sept. 1988, 18 Aug. 1989]

•***Lenzites warnieri*** Durieu and Mont., Annls Sci. Nat., Bot., sér. 4 14: 182 (1860)

Specimen examined: (49): on branch of *Populus nigra* L., 15 Jul. 1985, E. Krall, Z. Narbal, TAAM126870.

Literature: Baltaeva (1992, 1993), [(45): on wet branch of *Betula* sp., 17 Aug. 1987; (19): on *Populus* sp., 22 Jul. 1988; (7): died trunk of *Populus* sp. 10 Sept. 1988; (8): on *Salix* sp., 26 Sept. 1988; (26): on *Quercus* sp., 10 Aug. 1990; (18): on *Salix* sp., 18 Aug. 1988].

***Neolentinus lepideus*** (Fr.) Redhead and Ginns, Trans. Mycol. Soc. Japan 26(3): 357 (1985)

≡ *Lentinus lepideus* (Fr.) Fr.

Literature: Khalikova (1989), (as *Lentinus lepideus*, (28): on softwood conifer stumps, Sept. 1980, 1982); Baltaeva (1992), (as *Lentinus lepideus*, (45): on *Populus* sp. 13 Sept. 1990); Iminova (2009), (as *Lentinus lepideus*, (2): on stem of *Populus talassica* Kom., Nov. 2005).

***Perenniporia fraxinea*** (Bull.) Ryvarden, Grundr. Krauterk. 2: 307 (1978)

Literature: Baltaeva (1992), [(45): on stem of *Biota* sp., 13 Sept. 1990].

***Picipes badius*** (Pers.) Zmitr. and Kovalenko, International Journal of Medicinal Mushrooms (Redding) 18(1): 35 (2016)

≡ *Polyporus badius* (Pers.) Schwein

Literature: Baltaeva (1992), (as *Polyporus badius*, (19): on stump of woody plant, 13 Jul. 1989).

***Picipes melanopus*** (Pers.) Zmitr. and Kovalenko, International Journal of Medicinal Mushrooms (Redding) 18(1): 36 (2016)

≡ *Polyporus melanopus* (Pers.) Fr.

Literature: Baltaeva (1992), (as *Polyporus melanopus* (Pers.) Fr., (21): on *Prunus* sp., 2 Sept. 1990).

***Podofomes trogii*** (Fr.) Pouzar, Česká Mykol. 25(1): 19 (1971)

≡ *Ischnoderma trogii* (Fr.) Teixeira

Literature: Khalikova (1989), (as *Ischnoderma trogii*, (49): on *Abies alba* Mill., Oct. 1982).

***Polyporus lipsiensis*** (Batsch) E.H.L. Krause, Basidiomycetum Rostochiensium: 54 (1928)

≡ *Ganoderma lipsiense* (Batsch) G.F. Atk.

Literature: Iminova (2009), (as *Ganoderma lipsiense*, (6): on dried stem of angiosperm wood, Sept. 2002).

***Pycnoporus cinnabarinus*** (Jacq.) P. Karst., Revue mycol., Toulouse 3 (no. 9): 18 (1881)

Literature: Baltaeva (1992), [(45): on strum of *Salix* sp., 13 Jun. 1990].

***Pyrofomes demidoffii*** (Lév.) Kotl. and Pouzar, Reprium nov. Spec. Regni veg. 69: 140 (1964)

Specimen examined: (45): on living trunk of *Juniperus polycarpos* var. *seravschanica*, 29 Apr. 1988, I. Parmasto, TAAM126251.

Literature: Panfilova and Gaponenko (1963); Akhmedova (1966); Khalikova (1989), [(49): on living trunk of *Juniperus polycarpos* var. *seravschanica* and *Juniperus* sp.]; Baltaeva (1992, 1993), [(49): on trunk of living *Pinus* sp., 21 Jul. 1987; (45): on living Juniper trunk, 21 Jul. 1987; (8): on *Pinus* sp., 4 Jul. 1987; (7): on *Picea* sp., 25 Aug. 1989; (9): on *Pinus* sp., 14 Oct. 1987; (21): on *Pinus* sp., 14 Jul. 1987; (19): on *Picea* sp., 27 Jul. 1988].

*Note*: This species causes severe infections of living *Juniperus* trees in the study area.

***Szczepkamyces campestris*** (Quél.) Zmitr., Folia Cryptogamica Petropolitana (Sankt-Peterburg) 6: 52 (2018)

Literature: Baltaeva (1992, 1993), [(45): on broken trunk of *Quercus* sp., 11 Mar. 1985; (3): on *Pyrus* sp., 6 Jul. 1988; (19): on stem and branch of *Aesculus* sp., 15 May 1986; (7): on dried trunk of *Populus* sp., 21 Aug. 1989].

***Skeletocutis amorpha*** (Fr.) Kotl. and Pouzar, Česká Mykol. 12(2): 103 (1958)

Literature: Baltaeva (1992, 1993), [(45): on trunk of *Picea* sp., 15 Aug. 1987; (19): on *Picea* sp., 19 Aug. 1987; (19): on dried stem of *Abies alba*; (8): on trunk of *Pinus* sp., 8 Jul. 1988; (7): on *Abies alba*, 6 Aug. 1989; (21): on *Pinus* sp., 21 Aug. 1988].

***Skeletocutis nivea*** (Jungh.) Jean Keller, Persoonia 10(3): 353 (1979)

≡ *Incrustoporia nivea* (Jungh.) Ryvarden

Literature: Baltaeva (1992), (as *Skeletocutis nivea*), Baltaeva (1993), (as *Incrustoporia nivea*, (49): on wet branches of *Salix* sp., 10 Jun. 1988; (45): on stem of *Fraxinus* sp., 24 Jun. 1989).

#***Tinctoporellus epimiltinus*** (Berk. and Broome) Ryvarden, Trans. Br. mycol. Soc. 73(1): 18 (1979)

Specimen examined: (47): on decayed branches of *Populus* sp., 19 May 1990, K. Kalamees, M. Vaasma, TAAM144614.

*Note*: In TAAM, this specimen was incorrectly labeled as *Phellinus* sp.

***Trametes gibbosa*** (Pers.) Fr., Epicr. syst. mycol. (Upsaliae): 492 (1838) [1836–1838]

Literature: Baltaeva (1992, 1993), [(1): on wet trunk and stumps of deciduous woody plants: *Populus* sp., *P. tremula, Salix* sp., *Betula* sp., *Ulmus* sp.].

•***Trametes hirsuta*** (Wulfen) Lloyd, Mycol. Writ. 7(Letter 73): 1319 (1924)

Specimens examined: (28): on dried stem of living *Prunus vulgaris*, 3 Oct. 2011, YG312; (38): on dried unknown woody trunk, 1 Jun. 2011, YG073; (38): on unknown dried wood, 1 Jun. 2011, YG032; (38): on fallen trunk of angiosperm wood, 1 Jun. 2011, YG042; (38): on unknown decaying wood, 1 Jun. 2011, YG055; (40): on decaying *Juglans regia* log, 26 May 2011, YG002; (40): on trunk and branch of *Juglans regia*, 26 May 2011, YG037; (40): on *Prunus armeniaca*, 2 Nov. 2011, YG004; (40): on *Prunus vulgaris*, 2 Nov. 2011, YG007; (39): on living *Prunus* tree, 2 Nov. 2011, YG314; (37): on decaying, unidentified angiosperm stem, 19 Jun. 2014, YG/PS128; (37): on dead *Prunus* sp., 19 Jun. 2014, YG/PS138; (37): on dried stem of *Juglans regia*, 20 Jun. 2014, YG/PS168; (15): on died angiosperm strum, 9 Jul. 2017, RM44; (41): on stem of angiosperm tree, 14 Jul. 2014, YG/Ch40; (33): on fallen trunk of *Juglans regia*, 23 Apr. 1982, E. Parmasto, TAAM104394 in TAAM reported as *Antrodia* sp.

Literature: Panfilova and Gaponenko (1963), [(44): on *Prunus mahaleb*]; Khalikova (1989), [(49): on decaying branch of the *Quercus* tree]; Sinadskiy (1968), [(18): on fallen log of *Quercus*]; Baltaeva (1992, 1993), [(45): 1988, on stem of *Quercus* sp.; (21): on dried trunk of *Quercus* tree; (1): on various deciduous woody plant]; Iminova (2009), (as *Coriolus hirsutus* (Wulfen) Pat., (14): on fallen decaying branch of *Platanus orientalis* L. Jun. 2002; (14): on *Pinus brutia* var. *eldarica* (Medw.) Silba, May-Jun. 2002; 3: on dried trunk of *Prunus mahaleb*, Oct. 2003, *ibit*., (3): on dried on *Prunus mahaleb*, Nov. 2003).

*Note*: This is one of the most common and widespread species in Uzbekistan. This species is mostly found on *Prunus, Platanus, Pinus, Quercus*, and *Juglans* species in the study area.

***Trametes ochracea*** (Pers.) Gilb. and Ryvarden, N. Amer. Polyp., Vol. 2 Megasporoporia - Wrightoporia (Oslo): 752 (1987)

= *Trametes zonatella* Ryvarden

Specimen examined: (47): on fallen stems of *Prunus* sp., 17 May 1990, K.Kalamees, M. Vaasma, TAAM144571.

Literature: Panfilova and Gaponenko (1963); Kravtzev (1950); Khalikova (1989), (as *Trametes zonatella*, (49): on fallen branch of woody plants, Apr. 1985, May 1985); Iminova (2009), [(6): on unknown wood trunk, May 2000, Jul. 2005]; Baltaeva (1992), (as *Trametes ochracea*), Baltaeva (1993), (as *Trametes zonatella*, (1): on died trunks and stumps of deciduous woody plants); Iminova (2009), (as *Coriolus zonatus* (Nees) Quél., (2): on stump of woody plants, May, 2000, Jul. 2005).

***Trametes pubescens*** (Schumach.) Pilát, in Kavina and Pilát, Atlas Champ. l’Europe, III, Polyporaceae (Praha) 1: 268 (1939)

Literature: Baltaeva (1992), [(1): on died trunks, 1992].

***Trametes suaveolens*** (L.) Fr., Epicr. syst. mycol. (Upsaliae): 491 (1838) [1836–1838]

Literature: Baltaeva (1992, 1993), [(49): on trunk of *Salix* sp., 5 Sept. 1987; (45): on *Salix* sp., 16 Apr. 1987; (8): on *Populus tremula*, 11 May 1988; (19): on *Populus tremula*, 9 Jul. 1988; (21): on *Populus tremula*, 21 Sept. 1988].

***Trametes tephroleuca*** Berk. Hooker’s J. Bot. Kew Gard. Misc. 6: 165 (1854)

Specimens examined: (33): on fallen trunk of *Juglans regia*, 23 Apr. 1982, E. Parmasto, TAAM104394a, *ibit*. TAAM104399; (33): on fallen trunk of *Juglans regia*, 23 Apr. 1982, K. Kalamees, TAAM104305; (41): on a trunk of *Juglans regia*, 22 Apr. 1982, E. Parmasto, TAAM104270a; (33): on a trunk of *Lonicera* sp., 22 Apr. 1982, E. Parmasto, TAAM104283; (45): on dry twig of *Prunus* sp., 29 Apr. 1988, I. Parmasto, TAAM126249; (45): on dead trunk of *Prunus mahaleb*, 2 May 1988, A. Kollom, TAAM127624; (45): on fallen twig of *Prunus mahaleb*, 3 May 1988, I. Parmasto, TAAM126285; (45): on a dry trunk of *Crataegus* sp., 29 Apr. 1988, A. Kollom, TAAM127579; (41): on dry branch of *Crataegus pseudoheterophylla* subsp. *turkestanica*, 22 Aprel 1988, A. Kollom, TAAM127382.

Literature: Khalikova (1989), [(33): on dried stem of *Malus sieversii*, 17 Apr. 1986]; Iminova (2009), (as *Coriolus tephroleucus* (Fr.) Bonk., (14): on *Prunus vulgaris* and *Juglans regia*, May 1999).

•***Trametes trogii*** Berk., in Trog, Mittheil. d. schweiz. Naturf. Ges. in Bern 2: 52 (1850)

≡ *Funalia trogii* (Berk.) Bondartsev and Singer

Specimens examined: (28): on fallen deciduous branch, 2 Sept. 2013, YG/bot23b; 11 Sept. 2014; (38): on *Acer tataricum* subsp. *semenovii*, 9 Sept. 2016, YG1017; (49): on dried decaying trunk of *Populus alba*, 17 Sept. 2014, YG-N6, *ibit*. on trunk of *Populus alba*, 17 Sept. 2014, YG-N7; (31): on unidentified wood, 17 Sept. 2009, O. Kurina, TAAM189940 (as *Funalia* sp. in TAAM); (37): on dried on *Salix* sp. trunk, 14 Sept. 2014, YG/PS2X; (20): on *Populus* sp., 14 Sept. 2014, YG-JX4; (20): on unknown stump of angiosperm, 13 Jun. 2013, YG-G17, *ibit*. 13 Jun. 2013, YG-G18, *ibit*. 13 Jun. 2013, YG-G19; (20): on *Populus nigra* L., 16 Jun. 2013, YG-J4; (20): on dried stem of living *Populus nigra*, 15 Jun. 2013, YG-J6; (20): on *Populus* sp., 16 Jun. 2013, YG-GX1; (8): on unknown woody branches, 13 Jun. 2016, YG-G14, *ibit*., 13 Jun. 2016, YG1090.

Literature: Panfilova and Gaponenko (1963); Khalikova (1989), (as *Funalia trogii*, (30): on *Populus* sp.; (49): on unknown wood plant); Baltaeva (1992, 1993), [(45): on stem of *Salix* sp. 12 Sept. 1990; (1): mainly on dead stump and trunk of woody plants: on *Salix* sp., 27 Oct. 1992; (1): on stump and trunk of *Fraxinus* sp., 12 Sept. 1991; on fallen of *Populus tremula*, 15 Nov. 1992].

*Note*: First report of this species on stumps and trunks of *Acer tataricum* subsp. *semenovii*, and *Populus nigra* in Uzbekistan.

•***Trametes versicolor*** (L.) Lloyd, Mycol. Notes (Cincinnati) 65: 1045 (1921) [1920]

≡ *Coriolus versicolor* (L.) Quél.

Specimens examined: (28): on trunk of *Betula* sp., 5 May 1988, I. Parmasto, TAAM126293; (28): on *Betula* trunk, 5 May 1988, A. Kollom, TAAM127635; (37): on dried stem of angiosperm wood, 19 Jun. 2014, YG/PS128-1; (37): on dried stem of angiosperm wood, 19 Jun. 2014, YG/PS170; (45): on trunk of *Prunus* sp., 22 Apr. 1982, A. Kollom, TAAM127389; (49): on a fallen deciduous trunk, 22 Apr. 1982, A. Kollom, TAAM127388; (35): on *Crataegus pseudoheterophylla* subsp. *turkestanica* (Pojark.) K.I.Chr., 31 Aug. 1963, A. Raitviir, TAAM043489; (50): on trunk of *Lonicera* sp., 24 Apr. 1982, A. Kollom, TAAM127403; (45): on dry trunk of *Celtis australis* subsp. *caucasica*, 3 May 1988, I. Parmasto, TAAM126287, (45): on dry twig of *Prunus mahaleb*, 2 May 1988, I. Parmasto, TAAM126284; (47): on fallen *Prunus* branch, 17 May 1990, K. Kalamees, M. Vaasma, TAAM144572; (47): 4 May 1988, I. Parmasto, TAAM126294; (20): on *Prunus* sp., 14 Jun. 2013, YG-J3.

Literature: Panfilova and Gaponenko (1963); Akhmedova (1966); Baltaeva (1992, 1993); Khalikova (1989), [(1): on *Prunus mahaleb, Malus* sp., *Quercus* sp., *Juglans regia, Populus* sp.]; Iminova (2009), (as *Coriolus versicolor*, (12): on dried and living stem of *Prunus vulgaris*, May 2003, Jun. 2004).

*Note*: This species was recorded for the first time in Uzbekistan, on *Betula* sp., *Celtis australis* subsp. *caucasica, Crataegus pseudoheterophylla* subsp. *turkestanica*, and *Lonicera* sp. in Uzbekistan.

^∗^•***Trametes villosa*** (Sw.) Kreisel, Monografias, Ciencias, Univ. Habana, Ser. 4 16: 83 (1971)

Specimen examined: (29): on dried stem of angiosperm, 15 Jun. 2015, YG/AG11.

***Trametopsis cervina*** (Schwein.) Tomšovský, Czech Mycol. 60(1): 7 (2008)

≡ *Trametes cervina* (Schwein.) Bres.

Literature: Baltaeva (1992, 1993), (as *Trametes cervina*, (19): on fallen branch of *Juglans regia*, 14 May 1987; (7): on *Juglans regia*, 5 Jul. 1987; (45): on dried stem of *Morus alba*, 15 May 1988; (21): on stem of *Juglans regia*, 5 Jul. 1987; (3): on *Malus domestica*, 27 Mar. 1988; (26): *Morus alba* trunk, 15 May 1988; (26): on dried *Morus nigra*, 4 Aug. 1988; (9): on rotten trunk of *Morus alba*, 6 Aug. 1989).

*Note*: We could not observe the species in the localities mentioned in Baltaeva (1992, 1993).

***Tyromyces lacteus*** (Fr.) Murr, N. Amer. Fl. (New York) 9(1): 36 (1907)

Literature: Panfilova and Gaponenko (1963); Khalikova (1989), [(49): on died trunk of *Betula pendula* Roth].

SPARASSIDACEAE Herter

***Sparassis crispa*** (Wulfen) Fr., Syst. mycol. (Lundae) 1: 465 (1821)

Literature: Iminova (2009), [(3): on trunk of angiosperm trees, Aug. 2000, Sept. 2003, Oct. 2003].

GANODERMATACEAE Donk

•***Ganoderma adspersum*** (Schulzer) Donk, Proc. K. Ned. Akad. Wet., Ser. C, Biol. Med. Sci. 72(3): 273 (1969)

Specimens examined: (28): on died stump of *Acer saccharum* Marshall, 7 Jun. 2014, YG/bot24; (49): on strum of *Acer* sp., 12 Sept. 2011, YG/UG3; (49): on trunk of *Acer* sp., 12 Sept. 2014, YG/Gan1.

Literature: Gafforov (2014), [(28): on dead trunk of *Acer saccharum*, 14 Oct. 2011].

;*Note*: We found this species on decaying *Acer saccharum*, and this is the first report for *Ganoderma adspersum* on *Acer* from Central Asia. We collected *Ganoderma adspersum* only in Northeastern Uzbekistan. Outside of the study area, this species is mainly found in subatlantic or submediterranean regions and usually on trees such as *Tilia, Quercus, Fagus, Platanus*, and *Aesculus*.

***Ganoderma applanatum*** (Pers.) Pat., Hyménomyc. Eur. (Paris): 143 (1887)

Specimen examined: (23): on *Juglans regia*, 18 Aug. 2016, YG-B06.

Literature: Panfilova and Gaponenko (1963); Khalikova (1989), [(49): on stump of *Juglans regia*]; Schwartzman (1964), [(49): on dried stem of angiosperm woody plants], Akhmedova (1966), [(49): on *Populus* sp.]; Baltaeva (1992), [(1): on deadwood stem and stumps of deciduous trees]; Baltaeva, 1993 [(45): on dead fallen trunk of *Populus* sp., 16 Jul. 1986]; Iminova (2009), [(14): on various angiosperm trunks, Sept. 2002].

*Note*: This species is widespread in the study area and causes root rot disease of walnut trees.

***Ganoderma lucidum*** (Curtis) P. Karst., Revue mycol., Toulouse 3(no. 9): 17 (1881)

Literature: Panfilova and Gaponenko (1963); Schwartzman (1964); Khalikova (1989), [(49): on various deciduous wood]; Baltaeva (1992, 1993), [(49): on stump of deciduous wood, 13 Aug. 1989; (45): on living *Quercus* sp., 14 Aug. 1987; (19): on *Quercus* sp., 29 Jul. 1988; (19): on *Ulmus* sp., 2 Aug. 1988; (8): on stump of deciduous wood, 14 Aug. 1988; (6): on various deciduous woody plants, 9 Aug. 1989]; Iminova (2009), [(14): on trunks of angiosperm wood, 2000–2002].

#•***Ganoderma resinaceum*** Boud., in Patouillard, Bull. Soc. mycol. Fr. 5(2,3): 72 (1889)

Specimen examined: (8): on living stem of *Salix* sp., 7 Sept. 2016, YG-X4.

MERIPILACEAE Jülich

***Grifola frondosa*** (Dicks.) Gray, Nat. Arr. Brit. Pl. (London) 1: 643 (1821)

≡ *Polyporus frondosus* (Dicks.) Fr.

Literature: Iminova (2009), (as *Polyporus frondosus*, (3): on living *Juglans regia*, Jun. 2002, Jul. 2003).

FAMILY PLACEMENT UNCERTAIN (INCERTAE SEDIS)

^∗^•***Phlebiella christiansenii*** (Parmasto) K.H. Larss. and Hjortstam, in Hjortstam and Larsson, Mycotaxon 29: 316 (1987)

Specimens examined: (28): on fallen woody plant branch, 2 Sept. 2013, YG-G4; (28): on *Gleditsia triacanthos* L., 2 Sept. 2013, YG-G22; (28): on stem of fallen angiosperm tree, 2 Sept. 2013, YG-G26; (28): on dried stump of deciduous tree, 3 Sept. 2013, YG-G36; (38): on stump of *Juglans regia*, 3 Sept. 2013, YG-G040.

**HYMENOCHAETALES** Oberw.

HYMENOCHAETACEAE Imazeki and Toki

***Fomitiporia hippophaeicola*** (H. Jahn) Fiasson and Niemelä, Karstenia 24(1): 25 (1984)

≡ *Phellinus hippophaeicola* H. Jahn

Literature: Baltaeva (1992, 1993), (as *Phellinus hippophaeicola*, (49): on *Elaeagnus rhamnoides* (L.) A. Nelson, 26 Apr. 1989; (45): on *Elaeagnus rhamnoides*, 3 Sept. 1989; (21): on *Elaeagnus rhamnoides*, 3 Oct. 1989).

***Fomitiporia punctata*** (P. Karst.) Murrill, Lloydia 10: 254 (1947)

≡ *Phellinus punctatus* (P. Karst.) Pilát

Literature: Baltaeva (1992, 1993), (as *Phellinus punctatus*, (49): on *Ulmus* sp., 12 May 1988; (45): on *Crataegus* sp., 7 Aug. 1989; (45): on *Populus* sp., 21 Mar. 1987; (9): on *Populus* sp., 16 Apr. 1987; (21): on *Populus* sp., 9 Jul. 1987; (8): on *Ulmus* sp., 14 Aug. 1987; (8): on *Betula* sp., 21 Jul. 1989; (7): on *Ulmus* sp., 14 Aug. 1987; (19): on *Crataegus azarolus var. pontica* (K.Koch) K.I.Chr., 17 Apr. 1988).

***Fomitiporia robusta*** (P. Karst.) Fiasson and Niemelä, Karstenia 24(1): 25 (1984)

≡ *Phellinus robustus* (P. Karst.) Bourdot and Galzin

Literature: Panfilova and Gaponenko (1963); Khalikova (1989), (as *Phellinus robustus*, (49): on strums of *Spiraea* sp.); Baltaeva (1992, 1993), (as *Phellinus robustus*, (45): on stump of *Pistacia* sp., 7 Apr. 198; (20): on *Castanea* sp., 4 Mar. 1988; (19): on *Quercus* sp., 14 Mar. 1987; (21): on stem of *Quercus* sp., 24 Apr. 1987; (49): on *Populus* sp., 19 Aug. 1988; (8): on *Castanea* sp., 19 May 1987; (7): on *Juglans regia*, 30 Apr. 1988); Iminova (2009), (as *Phellinus robustus*, (16): on stem of *Ìorus alba*, Apr. 2001).

***Fulvifomes rimosus*** (Berk.) Fiasson and Niemelä, Karstenia 24(1): 26 (1984)

≡ *Phellinus rimosus* (Berk.) Pilát

Literature: Panfilova and Gaponenko (1963), (as *Phellinus rimosus*, (49): on trunk of *Pistacia vera*, 1963); Baltaeva (1992, 1993), (as *Phellinus rimosus*, (9): on *Quercus* trunk, 6 Jul. 1985; (21): on *Quercus*, 12 Jul. 1985; (19): on *Quercus* sp., 30 Jul. 1985; (45): on dried trunk of *Salix* sp., 25 Apr. 1986; (7): on *Salix* sp., 6 Jul. 1987; (8): on *Populus* sp., 19 Aug. 1989; (19): on *Populus* sp., 10 Apr. 1989).

***Fuscoporia contigua*** (Pers.) G. Cunn., Bull. N.Z. Dept. Sci. Industr. Res., Pl. Dis. Div. 73: 4 (1948)

≡ *Phellinus contiguus* (Pers.) Pat.

Literature: Baltaeva (1992, 1993), (as *Phellinus contiguus*, (21): on stem of *Elaeagnus rhamnoides*, 19 Sept. 1986; (45): on fallen *Acacia* trunk, 27 Aug. 1987, 6 Sept. 1987; (19): on stem of *Alnus* sp., 23 Sept. 1987; (7): on *Alnus* sp., 16 Aug. 1988; (8): on dried stem of *Ulmus* sp., 19 Sept. 1988; (9): on *Ulmus* sp., 30 Aug. 1989).

***Fuscoporia torulosa*** (Pers.) T. Wagner and M. Fisch., Mycol. Res. 105(7): 780 (2001)

≡ *Phellinus torulosus* (Pers.) Bourdot and Galzin

Literature: Khalikova (1989), (as *Phellinus torulosus* (Pers.) Bourdot and Galzin, (28): on living and died stems of *Quercus* sp., Jun.1986, Sept. 1987); Iminova (2009), (as *Phellinus torulosus*, (3): on *Betula tianschanica* Rupr., on *Salix babylonica* L., on *Pyrus communis* L., on *Morus nigra* L., Sept.–Oct. 1999–2003).

***Inocutis tamaricis*** (Pat.) Fiasson and Niemelä, Karstenia 24(1): 25 (1984)

≡ *Inonotus tamaricis* (Pat.) Maire

Literature: Gaponenko (1965); Sinadskiy and Bodartseva (1956), (as *Inonotus tamaricis*, (10): on living stem of *Tamarix hispida* Willd.); Baltaeva (1992, 1993), (as *Inonotus tamaricis*, (9): on *Tamarix ramosissima* Ledeb., 27 Aug. 1989; (9): on *Tamarix* sp., 24 Sept. 1986; (26): on *Tamarix* sp., 6 May 1987; (21): on stem of living *Tamarix hispida*, 31 Sept. 1987; (10): on dried stem of *Tamarix hispida*, 18 Mar. 1988).

***Inonotus andersonii*** (Ellis and Everh.) Černý, Česká Mykol. 17(1): 1 (1963)

Literature: Baltaeva (1992, 1993), [(45): on trunk of *Quercus* sp., 14 Aug. 1988, 2 Sept. 1989; (19): on stump of *Quercus* sp., 16 Sept. 1988].

***Inonotus cuticularis*** (Bull.) P. Karst., Meddn Soc. Fauna Flora fenn. 5: 39 (1879)

Literature: Baltaeva (1992), [(19): on *Juglans regia*, 10 Jul. 1990].

•***Inonotus hispidus*** (Bull.) P. Karst., Meddn Soc. Fauna Flora fenn. 5: 39 (1879)

Specimens examined: (40): on stem of living *Juglans regia*, 26 May 2011, YG054; (38): on *Juglans regia*, 6 Jun. 2011, YG035; (38): on stem of *Juglans regia*, 11 Jun. 2014, YG/UG1; (37): on living *Pinus* sp., 19 Jun. 2014, YG/PS156; (37): on trunk of living *Pinus* sp., 19 Jun. 2014, YG/PS157; (37): on *Malus sieversii*, 14 Sept. 2014, YG/PS148; (39): on living *Juglans regia*, 9 Sept. 2016, YG1015; (22): on *Juglans regia*, 11 Aug. 2015, YG-B07; (28): on dried trunk angiosperm wood, 27 Sept. 2014, YG/bot1; (29): on living *Morus alba* stem, 17 Sept. 2015, YG/AG1; (23): on living stem of *Juglans regia*, 15 May 2016, YG-B08; (41): on a wood of *Juglans regia*, 22 Apr. 1982, E. Parmasto, TAAM207844; (17): on a wood of *Morus alba*, 8 May 1976, K. Kalamees, TAAM080947.

Literature: Panfilova and Gaponenko (1963); Akhmedova (1966); Khalikova (1989); Baltaeva (1992, 1993); Iminova (2009), [(1): on living trunks of deciduous woody plants: *Malus domestica* Borkh., *M. sieversii* (Ledeb.) M.Roem., *Morus alba, Juglans regia, Prunus avium* (L.) L.].

***Inonotus obliquus*** (Fr.) Pilát, Atlas Champ. l’Europe, III, Polyporaceae (Praha) 1: 572 (1942)

Specimens examined: (28): unknown angiosperm fallen trunk, 14 Oct. 2011, YG001.

Literature: Baltaeva (1992, 1993), [(45): on living *Betula* sp., 28 Aug. 1987; (19): on stump of *Fraxinus* sp., 21 May 1986; (7): on *Alnus* sp., 9 Jul. 1986; (8): on dried fallen trunk of *Salix* sp., 13 Jun. 1987; (9): on *Salix* sp., 20 Apr. 1988; (3): on *Salix* sp., 29 Jun. 1989].

***Inonotus pseudohispidus*** Kravtzev, Bull. Acad. Sci. Kazakh SSR 98: 128 (1950)

Literature: Sinadskiy and Bodartseva (1956, 1960), (on living trunk of and *Populus pruinosa* Schrenk, *Populus euphratica* Oliv.); Baltaeva (1992, 1993), [(26): on *Populus* sp., 18 Jul. 1988; (9): trunk of *Populus* sp., 9 Aug. 1988; (21): on *Populus alba*, 20 Jul. 1989; (26): on *Populus* sp., 26 Aug. 1989].

***Mensularia radiata*** (Sowerby) Lázaro Ibiza, Revta R. Acad. Cienc. exact. fis. nat. Madr. 14(11): 736 (1916)

≡ *Inonotus radiatus* (Sowerby) P. Karst.

Literature: Baltaeva (1992, 1993), (as *Inonotus radiatus*, (6): on dried branch of *Alnus* tree, 3 Jul. 1986; (7): on stump of angiosperm woody plants, 19 May 1987; (8): on dried trunk and braches of *Ulmus* sp., 17 Jun. 1989; (45): on died trunk of *Quercus* sp., 7 Jul. 1987; (19): on *Quercus* sp., 17 Jun. 1987; (21): on *Ulmus* sp., 20 Aug. 1989).

***Phellinidium ferrugineofuscum*** (P. Karst.) Fiasson and Niemelä, Karstenia 24(1): 26 (1984)

≡ *Phellinus ferrugineofuscus* (P. Karst.) Bourdot and Galzin

Literature: Baltaeva (1992, 1993), (as *Phellinus ferrugineofuscus*, (45): on wood of *Pinus* sp., 15 Sept. 1988; (8): on *Pinus* sp., 24 Oct. 1988); (20): on stump of *Picea* sp., 6 Nov. 1987; (19): on *Picea* sp., 28 Oct. 1987).

***Phellinopsis conchata*** (Pers.) Y.C. Dai, Fungal Diversity 45: 309 (2010)

≡ *Phellinus conchatus* (Pers.) Quél.

Literature: Baltaeva (1992, 1993), (as *Phellinus conchatus*, (45): on dried stump *Syringa* sp., 10 Sept. 1988; (7): on *Populus* sp., 5 Oct. 1988; (6): on *Alnus* sp., 29 Aug. 1989; (21): on *Ulmus* sp., 13 Nov. 1988; (26): on dried stem of *Populus* sp., 21 Oct. 1988; (19): on *Alnus* sp., 8 Sept. 1989; (21): on *Ulmus* sp., 13 Oct. 1988).

^∗^•***Phellinus betulinus*** (Murrill) Parmasto, Folia cryptog. Estonica 43: 41 (2007)

Specimens examined: (50): on a trunk of *Betula tianschanica*, 24 Apr. 1982, E.Parmasto, TAAM104436; (50): on a dead branch of *Betula tianschanica*, 24 Apr. 1982, E.Parmasto, TAAM104285.

***Phellinus igniarius*** (L.) Quél., Enchir. fungi. (Paris): 177 (1886)

Specimen examined: (50): on a trunk of *Salix* sp., 24 Apr. 1982, A.Kollom, TAAM127406.

Literature: Panfilova and Gaponenko (1963); Khalikova (1989), [(41): on live trunk of *Juglans regia*, 1 Jun. 1980, Sept. 1984]; Baltaeva (1992, 1993), [(45): on trunk of *Prunus vulgaris*, 10 Sept. 1988; (21): on trunk of *Prunus* sp., 9 Aug. 1988; (9): on trunk of *Acer* sp., 12 Apr. 1987; (14): on *Acer* sp., 19 Aug. 1987; (8): on *Salix* sp., 6 Apr. 1988; (7): on *Salix* sp., 16 Jul. 1989; (19): on dried trunk of *Salix* sp., 24 Jul. 1988]; Iminova (2009), [(3): on *Juglans regia*, Apr.-May 2000].

*Note*: This species causes white rot of broad-leaved trees from many genera and is most common on *Alnus, Betula*, and *Corylus* spp. (all Betulaceae) and *Salix* spp. (Salicaceae), also fairly common on *Acer* (Sapindaceae) and *Malus, Prunus*, and *Sorbus* spp. (all Rosaceae), more rarely on *Aesculus, Amelanchier, Carpinus, Carya, Castanea, Cratageus, Fraxinus, Hippophae, Hydrangea, Juglans, Laburnum, Morus, Populus, Pterocarya, Robinia, Pyrus, Syringa, Tilia*, and *Ulmus*. Since this species is defined both in a wide and in a narrow sense, the lists of hosts should be interpreted with care.

•***Phellinus pomaceus*** (Pers.) Maire, Mus. barcin. Scient. nat. Op., Ser. Bot. 15: 37 (1933)

= *Phellinus tuberculosus* Niemelä

Specimens examined: (33): on a living trunk of *Prunus* sp., 26 Apr. 1982, E. Parmasto, TAAM104413; (35): on *Prunus cerasifera* Ehrh., 31 Aug. 1963, A. Raitviir, TAAM043492; (33): on a fallen trunk of *Prunus* sp., 23 Apr. 1982, A. Kollom, TAAM127401; (41): on the base living fruit trees, 26 Apr. 1982, E. Parmasto, TAAM104434; (50): on a trunk of *Prunus mahaleb*, 24 Apr. 1982, A. Kollom, TAAM127411; (50): on a dry trunk of *Prunus erythrocarpa* (Nevski) Gilli, 24 Apr. 1982, A. Kollom, TAAM203618; (50): on a trunk of *Salix* sp., 24 Sept. 2014, YG/S1; (38): on *Prunus cerasifera*, 1 Jun. 2011, YG052; (38): on *Prunus* sp., 11 Sept. 2011, YG51-ph; (38): on dried *Prunus* tree, 12 Sept. 2014, YG/Ug01; (38): on living *Prunus* sp., 12 Sept. 2014, YG/Ug02; (39): on dried stem of *Prunus dulcis* (Mill.) D.A.Webb, 2 Nov. 2011, YG009; (39): on living stem of *Cerasus tianshanica* Pojark., 2 Nov. 2011, YG028, *ibit* on living stem of *Cerasus tianshanica* Pojark., 20 Sept. 2014, YG/PS3X; (39): on *Prunus cerasifera*, 2 Nov. 2011, YG337; (39): on living trunk of *Prunus cerasifera*, 2 Nov. 2011, YG338; (32): on *Prunus mahaleb*, 15 May 2011, YG28; (32): on dried trunk of *Prunus* sp., 13 Sept. 2014, YG/bil164; (37): on *Prunus* sp., 19 Sept. 2014, YG/PS82; (45): on a dry trunk of *Crataegus altaica* Ledeb., 2 May 1988, I. Parmasto, TAAM126247; (48): on *Lonicera* sp., Sept. 1982, N.I. Gaponenko, TASM582; (8): on dried stem of *Prunus* sp., 9 Sept. 2016, YG1102; (45): on dry branch of *Juglans regia*, 29 Apr. 1988, I. Parmasto, TAAM126253; (45): on dry trunk of *Celtis australis* subsp. *caucasica*, 1 May 1988, I. Parmasto, TAAM126269. In TAAM two specimens reported as *Phellinus* sp.

Literature: Panfilova and Gaponenko (1963); Khalikova (1989), (as *Phellinus tuberculosus*, (49): on trunks of Rosaceae family trees); Baltaeva (1992, 1993), (as *Phellinus tuberculosis*, (49): on *Prunus* spp., 10 May 1987; (45): on *Malus domestica*, 13 May 1987; (8): on *Malus* sp., 16 May 1989; (19): on *Prunus* sp., 26 May 1987); Iminova (2009), (as *Phellinus pomaceus*, (2): on *Prunus persica* (L.) Batsch, Apr. 2004); Iminova (2009), (as *Phellinus tuberculosis*, (15): on living stem of *Cydonia oblonga* Mill., May 2003; (2): on trunk of living *Prunus domestica* L. and on *Malus domestica*, May–Aug. 2002).

*Note*: First report of this species is on *Celtis australis* subsp. *caucasica* and *Lonicera* sp. from Uzbekistan. Usually it is largely confined to trees belonging to the Rosaceae, chiefly on *Prunus* and rarely on *Malus, Pyrus*, and *Cydonia*, and causes white rot of the heartwood of living fruit trees. This species is widespread in the northern hemisphere and probably occurs wherever native species of *Prunus* from the stone fruit group and where peaches, cherries, and plums are cultivated as fruit trees. This species is also reported on *Acer, Alnus, Carpinus, Ceratonia, Cornus, Corylus, Crataegus, Fagus, Ficus, Juglans, Malus, Olea, Pyrus, Salix*, and *Ulmus*.

***Phellinus tremulae*** (Bondartsev) Bondartsev and P.N. Borisov, Trut. Grib Evrop. Chasti SSSR Kavkaza [Bracket Fungi Europ. U.S.S.R. Caucasus] (Moscow-Leningrad): 358 (1953)

Literature: Baltaeva (1992, 1993), [(45): on trunk of *Populus tremula*, 20 Jul. 1985; (19): on *Populus tremula*, 16 Aug. 1985; (21): on *Populus tremula*, 10 Aug. 1986; (7): on *Populus* sp., 25 Aug. 1986; (26): on dried fallen of *Populus tremula*, 19 Aug. 1987; (6): on living of *Populus* sp., 8 Aug. 1988]; Khalikova (1989), [(49): on live trunk of *Populus* sp., 20 Jul. 1985].

***Phylloporia ampelina*** (Bondartsev and Singer) Bondartseva, Mikol. Fitopatol. 17(4): 279 (1983)

≡ *Phellinus ampelinus* Bondartsev and Singer

Literature: Bondarceva and Parmasto (1986), (as *Phellinus ampelinus*, (49): on dead and live trunk of *Vitis vinifera* L.).

^∗^***Phylloporia ephedrae*** (Woron.) Parmasto, Proc. Indian Acad. Sci., Pl. Sci. 94(2–3): 377 (1985)

Specimens examined: (45): on stem of living *Ephedra equisetina* Bunge, 1 May 1988, I. Parmasto, TAAM126265; (45): on *Ephedra equisetina*, 2 May 1988, I. Parmasto, TAAM126279; (45): on stem of *Ephedra equisetina*, 1 May 1988, A. Kollom, TAAM127593.

***Phylloporia yuchengii*** Gafforov, Tomšovský, Langer and L.W. Zhou, Cryptog. Mycol. 35(4): 318 (2015) [2014]

Specimens examined: (38): on trunk of *Juglans regia*, 1 Jun. 2011, YG043; (40): on *Prunus* sp., 11 Sept. 2011, YG343; (45): on a trunk of *Juglans regia*, 29 Apr. 1988, I. Parmasto, TAAM126260, in TAAM as *Phellinus* sp.; (7): on trunk of *Crataegus pseudoheterophylla* subsp. *turkestanica*, 11 Sept. 2015, YG1093; (39): on *Crataegus* sp., 9 Oct. 2016, YG1011; (8): on fallen unknown woody branches, 9 Sept. 2016, YG1101; (20): on trunk of *Populus* sp., 12 Jun. 2013, YG-J5; (20): on *Morus alba*, 13 Jun. 2013, YG-J10; (20): on *Morus alba*, 13 Jun. 2013, YG-J11.

Literature: Gafforov et al. (2014), [(38): on dead angiosperm trunk and stem, 1 Jun. 2011; (39): on dead angiosperm trunk, 2 Nov. 2011].

*Note*: This species was first described from northeastern Uzbekistan in 2014. Later, we collected this species in central and south Uzbekistan. It seems that this species is widespread in the study area. This species grows on *Crataegus, Juglans, Morus, Populus*, and *Prunus*, which represent four plant families.

***Porodaedalea pini*** (Brot.) Murrill, Bull. Torrey bot. Club 32(7): 367 (1905)

≡ *Phellinus pini* (Brot.) Pilát

Literature: Baltaeva (1992, 1993), (as *Phellinus pini*, (1): on trunks and stumps of conifer tees); Khalikova (1989), (as *Phellinus pini*, (28): on live trunk of *Pinus pallasiana* D. Don, Oct. 1984); Iminova (2009), (as *Phellinus pini*, (13): on dried stem of angiosperm, May 2003).

•***Sanghuangporus lonicerinus*** (Bondartsev) Sheng H. Wu, L.W. Zhou and Y.C. Dai, in Zhou, Vlasák, Decock, Assefa, Stenlid, Abate, Wu and Dai, Fungal Diversity 77: 340 (2015)

≡ *Phellinus lonicerinus* (Bondartsev) Bondartsev and Singer

Specimens examined: (49): on dried stem of *Lonicera* sp., 8 Nov. 2016, YG1095; (36): on *Lonicera* sp., 8 Nov. 2016, YG1096; (32): on stem of living *Lonicera nummulariifolia* Jaub. and Spach, 15 May 2011, YG018; (41): on the base of a living trunk of *Lonicera* sp., 22 Apr. 1982, E. Parmasto, TAAM203688; (36): on a trunk of *Lonicera* sp., 25 Apr. 1982, E. Parmasto, TAAM104407; (36): on the base of a living trunk of *Lonicera* sp., 25 Apr. 1982, E. Parmasto, TAAM104439; (50): on at the base of *Lonicera* sp., 24 Apr. 1982, E. Parmasto, TAAM0104264; (50): on a dry trunk of *Lonicera* sp., 24 Apr. 1982, A. Kollom, TAAM127410; (39): on *Lonicera* sp., 9 Oct. 2016, YG1012; (50): on *Lonicera nummulariifolia*, 9 Oct. 2016, YG1013; (38): on *Lonicera* sp., 9 Sept. 2016, YG1016; (37): 19 Jun. 2014, on living stem of *Lonicera* sp., YG/PS92; (37): 20 Jun. 2014, dried stem of *Lonicera* sp., YG/PS129; (37): on *Acer* sp., 20 Jun. 2014; (30): on living stem of *Acer tataricum* subsp. *semenovii*, 5 May 2014, YG/Un1; (46): on a dry trunk of deciduous trunk, 29 Apr. 1988, A. Kollom, TAAM127578; (7): on *Lonicera* sp., 11 Sept. 2016, YG1094; (8): on *Lonicera* sp., 26 May 2018, YG1097; (8): on living stem of *Lonicera microphylla* Willd. ex Schult., 26 May 2018, YG1112.

Literature: Panfilova and Gaponenko (1963); Khalikova (1989); Iminova (2009), (*Phellinus lonicerinus*, (1): on *Lonicera* spp.).

*Note*: This species was thought to grow exclusively on *Lonicera*, but a new host *Acer tataricum* subsp. *semenovii* is recorded here from Uzbekistan.

***Tropicoporus linteus*** (Berk. and M.A. Curtis) L.W. Zhou and Y.C. Dai, in Zhou, Vlasák, Decock, Assefa, Stenlid, Abate, Wu and Dai, Fungal Diversity 77: 344 (2015)

≡ *Phellinus linteus* (Berk. and M.A. Curtis) Teng

Specimen examined: (42): on dry branch of *Rosa fedtschenkoana* Regel, 21 Apr. 1982, E. Parmasto, TAAM104272 (as *Phellinus* sp. in TAAM).

Literature: Panfilova and Gaponenko (1963); Khalikova (1989), (as *Phellinus linteus*, (49): on dead and living trunk and stem of angiosperm woody plants); Baltaeva (1992, 1993), (as *Phellinus linteus*, (49): on *Salix* sp., 16 Aug. 1985, (45): on living trunk of *Lonicera* sp., 9 Apr. 1985; (21): on *Quercus* sp., 12 Sept. 1986; (7): on *Populus* sp., 5 May 1987; (8): on *Acer* sp., 4 Sept. 1989; (19): on *Ulmus* sp., 27 Aug. 1987); Iminova (2009), (as *Phellinus linteus*, (3): on *Salix wilhelmsiana*, Apr. 2000, May 2003).

NEOANTRODIELLACEAE Y.C. Dai, B.K. Cui, Jia J. Chen and H.S. Yuan

¤•***Neoantrodiella*** sp.

Specimen examined: (33): on trunk of *Juniperus polycarpos* var. *seravschanica*, 22 Apr. 1982, E. Parmasto, TAAM104307.

OXYPORACEAE Zmitr. and Malysheva

***Rigidoporus corticola*** (Fr.) Pouzar, Folia geobot. phytotax. bohemoslov. 1(4): 368 (1966)

≡ *Oxyporus corticola* (Fr.) Ryvarden

Specimens examined: (49): on fallen trunk, 22 Sept. 2014, YG-P55, *ibit*., YG-P67; (49): on dried branch of angiosperm, 23 Sept. 2014, YG-P35.

Literature: Baltaeva (1992, 1993), (as *Oxyporus corticola*, (21): on stump of *Quercus* sp., 6 May, 1985; (21): on *Fraxinus* sp., 12 Aug. 1988; (45): on stem of *Fraxinus* sp., 6 May 1988; (20): on dried stem of living *Salix alba*, 29 Aug. 1989; (19): on dead trunk of *Populus* sp., 15 Apr. 1987; (9): on *Populus* sp., 30 Jul. 1987; (7): on *Populus tremula*, 19 Oct. 1987).

***Rigidoporus latemarginatus*** (Durieu and Mont.) Pouzar, Folia geobot. phytotax. bohemoslov. 1(4): 368 (1966)

≡ *Oxyporus latemarginatus* (Durieu and Mont.) Donk

= *Chaetoporus ambiguus* (Bres.) Bondartsev and Singer

Literature: Baltaeva (1992), (as *Oxyporus latemarginatus*, (28): on stump of *Pyrus* sp., 16 Sept. 1990), Sinadskiy and Bodartseva, 1960 (as *Chaetoporus ambiguus*, (27): on *Elaeagnus rhamnoides*, Jun. 1960).

***Rigidoporus populinus*** (Schumach.) Pouzar, Folia geobot. phytotax. bohemoslov. 1(4): 368 (1966)

≡ *Oxyporus populinus* (Schumach.) Donk

Literature: Panfilova and Gaponenko (1963), (as *Oxyporus populinus*, 49: on dead stem of *Acer tataricum* L.), Khalikova (1989), (as *Oxyporus populinus*, (49): on fallen branch and stem *Populus* sp., May 1987; on *Quercus* sp., Jun. 1988); Baltaeva (1992, 1993), (as *Oxyporus populinus*, (1): on dried stem of living *Sorbus* sp.).

•^∗^***Rigidoporus ginkgonis*** (Y.C. Dai) F. Wu, Jia J. Chen and Y.C. Dai, in Wu, Chen, Ji, Vlasák and Dai, Mycologia 109(5): 761 (2017)

Specimens examined: (28): on fallen trunk and stump of deciduous woody plants, 2 Sept. 2013, YG-G2, *ibid*. on fallen trunk of deciduous woody plants, YG-G3; (28): on decaying branch of angiosperm, 3 Sept. 2013, YG-G35.

SCHIZOPORACEAE Jülich

***Hyphodontia alutaria*** (Burt) J. Erikss., Symb. bot. upsal. 16(no. 1): 104 (1958)

Literature: Gafforov et al. (2017), [(28): on twigs and stem of *Pterocarya pterocarpa* Kunth ex I. Iljinsk, 2 Sept. 2013].

#***Hyphodontia arguta*** (Fr.) J. Erikss., Symb. bot. upsal. 16(no. 1): 104 (1958)

Specimen examined: (49): on fallen tree, 21 Oct. 2015, YG-X66.

•***Hyphodontia zhixiangii*** L.W. Zhou and Gafforov, Phytotaxa 299(2): 275 (2017)

Specimens examined: (8): on unknown angiosperm branch, 9 Sept. 2016, YG1098; (49): on fallen angiosperm branches, 7 Oct. 2016, YG1104.

Literature: Kan et al., 2017 [(8): on stem of *Juniperus* sp., 9 Sept. 2016].

***Lyomyces crustosus*** (Pers.) P. Karst., Revue mycol., Toulouse 3(no. 9): 23 (1881)

Literature: Gafforov et al. (2017), [(38): on branch of living tree *Fraxinus pennsylvanica* Marshall, 3 Sept. 2013].

•***Lyomyces erastii*** (Saaren. and Kotir.) Hjortstam and Ryvarden, Syn. Fung. (Oslo) 26: 43 (2009)

Specimen examined: (15): on unknown shrub, 12 Jul. 2017, RM21.

Literature: Gafforov et al. (2017), [(39): on deciduous wood, 2 Nov. 2011].

***Lyomyces sambuci*** (Pers.) P. Karst., Bidr. Känn. Finl. Nat. Folk 37: 153 (1882)

Literature: Gafforov et al. (2017), [(28): on dead wood of *Philadelphus* sp., 20 Apr. 1982; (39): on a dead branch of angiosperm, 2 Sept. 2013].

***Xylodon paradoxus*** (Schrad.) Chevall., Fl. gén. env. Paris (Paris) 1: 274 (1826)

≡ *Schizopora paradoxa* (Schrad.) Donk

Literature: Baltaeva (1992, 1993), (as *Schizopora paradoxa*, (49): on trunks of *Quercus* sp., 20 Aug. 1989; (45): on trunk of *Quercus* sp., 7 Oct. 1988; (8): on *Quercus* sp., 19 Nov. 1989).

TAXA WITH UNCERTAIN POSITION AT THE FAMILY LEVEL (INCERTAE SEDIS)

***Sidera lenis*** (P. Karst.) Miettinen, in Miettinen and Larsson, Mycol. Progr. 10(2): 136 (2011)

≡ *Diplomitoporus lenis* (P. Karst.) Gilb. and Ryvarden

≡ *Antrodia lenis* (P. Karst.) Ryvarden

Literature: Baltaeva (1992), (as *Diplomitoporus lenis*), Baltaeva, 1993 (as *Antrodia lenis*, (45): on trunk of *Pinus* sp., 8 Jul. 1988; (8): on *Pinus* sp., 6 Jul. 1989; (19): on wet of trunk of *Picea* sp., 27 Aug. 1989; (19): on *Picea* sp., 14 Jul. 1987); Khalikova (1989), (as *Antrodia lenis*, (49): on living *Juniperus* tree, 17 Jul. 1988).

***Trichaptum abietinum*** (Pers.) Ryvarden, Norw. Jl Bot. 19: 237 (1972)

Literature: Baltaeva (1992, 1993), [(45): on wet trunk of *Pinus* sp., 12 Mar. 1988; (7): on stump of *Pinus* sp., 15 Aug. 1988; (19): on *Pinus* sp., 9 Jul. 1989].

***Trichaptum biforme*** (Fr.) Ryvarden, Norw. Jl Bot. 19(3–4): 237 (1972)

= *Hirschioporus pergamenus* (Fr.) Bondartsev and Singer

Literature: Panfilova and Gaponenko (1963), (as *Hirschioporus pergamenus*, (49): on dried woody plants); Khalikova (1989), [(49): on dead trunk of angiosperms tree]; Baltaeva (1992, 1993), [(45): on fallen stem of *Populus* sp., 18 Jul. 1988; (26): on *Salix* sp., 18 Jul. 1988; 9: on dead trunk of *Populus* sp., 7 Sept. 1987; (21): on *Salix* sp., 15 Sept. 1987].

***Trichaptum fuscoviolaceum*** (Ehrenb.) Ryvarden, Norw. Jl Bot. 19: 237 (1972)

Specimen examined: (49): on a fallen trunk of *Abies sibirica* Ledeb., 29 Aug. 1958, E. Parmasto, TAAM009360.

Literature: Baltaeva (1992), [(45): on stump of *Pinus* sp., 17 Aug. 1990].

*Note*: This species is reported for the first time on *Abies sibirica* from Uzbekistan.

**RUSSULALES** Kreisel ex P.M.Kirk, P.F.Cannon and J.C.David

BONDARZEWIACEAE Kotl. and Pouzar

***Heterobasidion annosum*** (Fr.) Bref., Unters. Gesammtgeb. Mykol. (Liepzig) (8): 154 (1888)

Literature: Baltaeva (1992, 1993), [(49): on *Picea* sp., 10 Sept. 1988, 30 Jul. 1989; (45): on root of *Biota* sp., 15 Nov. 1987; (19): on stump of *Pinus* sp., 6 Sept. 1988; (7): on *Picea* sp., 10 Sept. 1988].

LACHNOCLADIACEAE D.A. Reid

#***Vararia parmastoi*** Boidin and Lanq., Persoonia 12(3): 257 (1984)

Specimens examined: (50): on decayed trunk of *Juniperus semiglobosa*, 24 Apr. 1982, E. Parmasto, TAAM104302 (Paratype); (50): on dead roots of *Juniperus semiglobosa*, 24 Apr. 1982, E. Parmasto TAAM104303 (Isotype); (50): on a fallen rotten trunk of *Juniperus semiglobosa*, 24 Apr. 1982, E. Parmasto, TAAM104294; (36): on trunk of *Juniperus semiglobosa*, 25 Apr. 1982, E. Parmasto, TAAM104440.

PENIOPHORACEAE Lotsy

^∗^***Peniophora cinerea*** (Pers.) Cooke, Grevillea 8(no. 45): 20 (1879)

Specimens examined: (39): on fallen trunks of angiosperm woody plant, 2 Nov. 2011, YG039; (39): on stem and dried branch of *Juglans regia*, 1 Jun. 2011, YG058.

^∗^***Peniophora incarnata*** (Pers.) P. Karst., Hedwigia 28: 27 (1889)

Specimen examined: (39): on fallen stem of deciduous wood, 16 Jun. 2014, YG/PS84.

STEREACEAE Pilát

^∗^***Stereum gausapatum*** (Fr.) Fr., Hymenomyc. eur. (Upsaliae): 638 (1874)

Specimen examined: (49): on trunk of *Picea schrenkiana* Fisch. and C.A.Mey., 7 Sept. 2013, YG-Gxx.

*Note*: This species occurs on *Quercus, Castanea*, and *Carpinus* species in Europe, particularly in the Mediterranean area. Fruiting bodies develop on dead stems, rotten stumps, trunks, or more rarely on fallen branches and other debris of angiosperm woody plants. However, we found that species on the conifer tree, *Picea schrenkiana* from Western Tien Shan Mountains of Uzbekistan.

***Stereum hirsutum*** (Willd.) Pers., Observ. mycol. (Lipsiae) 2: 90 (1800) [1799]

Specimens examined: (33): on deciduous tree trunk, 23 Apr. 1982, E. Parmasto, TAAM104393; (43): on unknown woody branch, 8 Sept. 2016, YG1091; (43): on fallen trunk of angiosperm wood, 8 Sept. 2016, YG1092; (40): on dried stem of *Juglans regia*, 26 May 2011, YG029; (46): on *Crataegus* sp., 13 May 1990, K. Kalamees and M. Vaasma, TAAM144492; (32): on *Acer tataricum* subsp. *semenovii*, 15 May 2011, YG030; (32): on *Acer* sp., 15 May 2011, YG51; (32): on unknown decaying wood, 15 May 2011 YG056; (32): on *Acer tataricum* subsp. *semenovii*, 15 May 2011, YG057; (32): on unknown dried wood, 7 Jun. 2011 YG048; (39): on stem of living *Salix iliensis* Regel, 2 Nov. 2011, YG034; (38): on *Juglans regia*, 1 Jun. 2011, YG109; (38): on dried stem of *Fraxinus excelsior* L., 3 Sept. 2013, YG-G12; (38): on *Quercus* sp., 3 Sept. 2013, YG-G15; (38): on dried stem of *Salix alba*, 3 Sept. 2013, YG320; (37): on living stem of *Juglans regia*, 19 Jun. 2014, YG/PS135; (37): on dried trunk of *Juglans regia*, 19 Jun. 2014, YG/PS176; (45): on a dry trunk of *Celtis australis* subsp. *caucasica*, 29 Apr. 1989, TAAM126246; (45): on *Crataegus pseudoheterophylla* subsp. *turkestanica*, 29 Apr. 1988, I. Parmasto, TAAM126255; (45): on trunk of *Quercus* sp., 29 Apr. 1988, A. Kollom, TAAM127585; (46): on trunk of *Populus alba*, 4 May 1988, I. Parmasto, TAAM126291 (as *Coriolopsis* sp.); (8): on fallen branch, 10 Sept. 2016, YG1099; (28): on dead fallen trunk of angiosperm, 3 Apr. 2013, YG3.04.13.

Literature: Panfilova and Gaponenko (1963), [(30): on *Populus* sp.]; Khalikova (1989), [(28): on dried branch of living *Quercus robur* L.]; Iminova (2009), [(14): on *Salix interior*, Apr. 2002; (3): on *Juglans regia*, May 2005].

^∗^***Stereum rugosum*** Pers., Neues Mag. Bot. 1: 110 (1794)

Specimen examined: (50): on a dead trunk of *Salix* sp., 24 Apr. 1982, TAAM127402

*Note*: This species is very common and widespread on moist various deciduous and coniferous forests dead trees in the northern hemisphere especially in Europe (Breitenbach and Kränzlin, 1986). It is rarely reported from East and South Asia (Dai, 2011). This is the first report of the species from the mountainous area of Central Asia.

^∗^***Stereum subtomentosum*** Pouzar, Ćeská Mykol. 18(3): 147 (1964)

Specimen examined: (28): on dead trunk of angiosperm, 24 Sept. 2014, YG-P37.

*Note*: This species is rare in the study area and generally reported in Betulaceae (*Alnus, Betula, Carpinus*) and Salicaceae (*Populus, Salix*) plant species from Europe. It is easily distinguished when fresh by the distinct yellowish exudate when cut. It is similar to *Stereum ostrea*, which, however, has a tropical or subtropical distribution and is distinguished by its oyster-like fruitbody and more brownish color.

**THELEPHORALES** Corner ex Oberw.

BANKERACEAE Donk

***Phellodon fuligineoalbus*** (J.C. Schmidt) R.E. Baird, in Baird, Wallace, Baker and Scruggs, Fungal Diversity 62: 63 (2013)

≡ *Bankera fuligineoalba* (J.C. Schmidt) Coker and Beers.

Literature: Schwartzman (1964), (as *Bankera fuligineoalba* (49): on *Salix caprea* L., and on *Betula pendula*).

***Sarcodon imbricatus*** (L.) P. Karst., Revue mycol., Toulouse 3(no. 9): 20 (1881)

= *Sarcodon squamosus* (Schaeff.) Quél.

Literature: Schwartzman (1964), (as *Sarcodon squamosus* (49): on old stump of *Populus tremula*).

THELEPHORACEAE Chevall.

#***Pseudotomentella mucidula*** (P. Karst.) Svrćek, Ćeská Mykol. 12(2): 68 (1958)

Specimen examined: (47): on fallen trunk of *Pinus sibirica* Du Tour, 29 Aug. 1958, TAAM009363.

**TRECHISPORALES** K.H. Larss.

HYDNODONTACEAE Jülich

***Fibuloporia desertorum*** (Kravtzev) Schwartzman, Flora Sporovykh Rastenii Kazakhstana [Cryptogamic Flora of Kazakhstan], 4, Auriculariales, Tremellales, Dacryomycetales, Exobasidiales, Aphyllophorales (Alma-Ata): 299 (1964)

≡ *Dextrinosporium desertorum* (Kravtzev) Bondartseva

Literature: Gaponenko (1965), (as *Dextrinosporium desertorum*, (10): on living *Haloxylon persicum*).

**AGARICOMYCETES Doweld**

ORDER AND FAMILY UNCERTAIN (INCERTAE SEDIS)

^∗^•***Peniophorella praetermissa*** (P. Karst.) K.H. Larss., Mycol. Res. 111(2): 192 (2007)

Specimens examined: (38): on living stem of *Juglans regia*, 3 Sept. 2013, YG-G16; (38): on fallen unknown angiosperm branches, 3 Sept. 2013, YG-G40; (28): on dried stump of deciduous tree, 3 Sept. 2013, YG-G37.

### Substrate Preferences of Wood-Inhabiting Poroid and Corticioid Species in Uzbekistan

**FIGURE 8:**
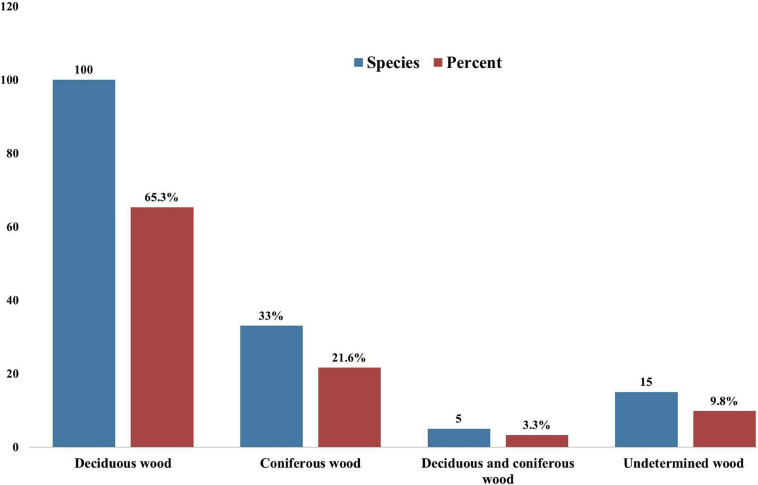
Species richness and proportions of wood-inhabiting poroid and corticioid fungi on different wood types.

In this study, poroid and corticoid fungal species were found on 100 woody plant species belonging to 23 families and 42 genera. One hundred wood-inhabiting species (accounting for 65.3% of the total wood-inhabiting poroid and corticoid species of Uzbekistan) were recorded exclusively on deciduous wood, 33 species (21.6%) were found exclusively on coniferous wood, and 5 species were recorded as inhabiting both groups of woody plants (Figure 8). The hosts were not determined for the remaining 15 species. These wood-inhabiting fungi were most frequently found on hosts belonging to Salicaceae (72 fungal species), Pinaceae (51), Rosaceae (48), Fagaceae (31), Juglandaceae (30), Betulaceae (25), Cupressaceae (16), Sapindaceae (16), and Ulmaceae and Oleaceae (each 11). Collectively, these families host about 70% of fungal species present in the study area; other plant families host one to nine species (Table 3).

**TABLE 3:** Number of host family, genus, and species and number of wood-inhabiting poroid and corticioid species on host family in the study area.

Host family	No. of host genera	No. of host species	No. of fungal species
Salicaceae	2	16	72
Pinaceae	3	8	51
Rosaceae	9	27	48
Fagaceae	2	3	31
Juglandaceae	2	2	30
Betulaceae	2	4	25
Cupressaceae	2	5	16
Sapindaceae	2	6	16
Ulmaceae	1	1	11
Oleaceae	2	5	11
Moraceae	1	2	9
Caprifoliaceae	1	4	8
Cannabaceae	1	1	7
Leguminosae	3	3	6
Anacardiaceae	1	2	3
Platanaceae	1	1	3
Elaeagnaceae	1	1	3
Amaranthaceae	1	2	2
Tamaricaceae	1	3	1
Vitaceae	1	1	1
Ephedraceae	1	1	1
Hydrangeaceae	1	1	1
Malvaceae	1	1	1
Total: 23	42	100	n.a.

**FIGURE 9:**
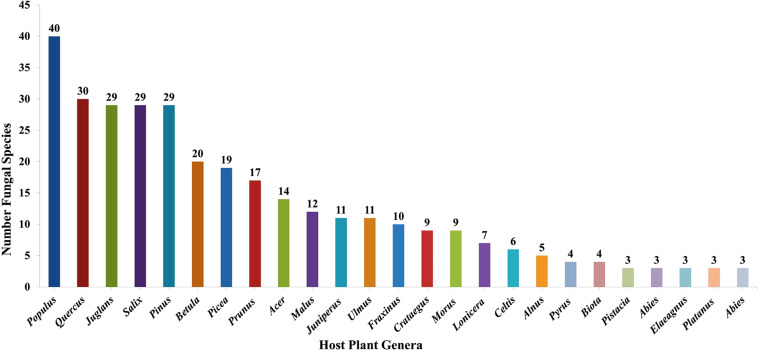
Occurence numbers of wood-inhabiting poroid and corticioid fungi on most representative host genera.

The highest number of wood-inhabiting poroid and corticoid species is reported in the following host genera: *Populus* (40 species, 26.4% of the total species number), *Quercus* (30, 19.6%), *Juglans, Pinus*, and *Salix* (each 29, 18.9%), *Betula* (20, 13.0%), *Picea* (19, 12.4%), *Prunus* (17, 11.15%), *Acer* (14, 9.2%), *Malus* (12, 7.8%), *Juniperus* and *Ulmus* (each 11, 7.1%), *Fraxinus* (10, 6.5%); other plant genera host one to nine fungal species (Figure 9).

**FIGURE 10:**
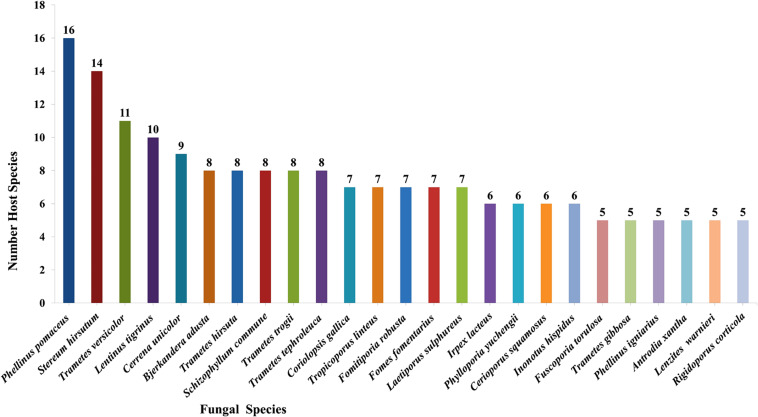
Host species numbers of 25 wood-inhabiting poroid and corticioid species with the widest host range.

Among the wood-inhabiting poroid and corticioid fungi, 25 species were associated with a wide range of plant hosts, such as *Phellinus pomaceus* (16 host species), *Stereum hirsutum* (14), *Trametes versicolor* (11), *Lentinus tigrinus* (10), *Cerrena unicolor* (9), *Bjerkandera adusta, Schizophyllum commune, Trametes hirsuta, T. tephroleuca*, and *T. trogii* (8 hosts each); *Coriolopsis gallica, Fomes fomentarius, Fomitiporia robusta, Tropicoporus linteus*, and *Laetiporus sulphureus* (7 each); *Irpex lacteus, Phylloporia yuchengii, Inonotus hispidus*, and *Cerioporus squamosus* (6 each); and *Fuscoporia torulosa, Trametes gibbosa, Antrodia xanthan, Phellinus igniarus, Lenzites warnieri*, and *Rigidoporus corticola* (5 each) (Figure 10). The other 128 wood-inhabiting poroid and corticioid fungi studied each grew on one to four host species. Regarding the host preference, some wood-inhabiting poroid and corticioid species are tolerant, such as *Phellinus pomaceus* recorded on 16 plant species of nine genera (*Prunus* sp., *P. mahaleb, P. cerasifera, P. erythrocarpa, P. dulcis, P. persica, P. domestica, Cerasus tianshanica, Cydonia oblonga, Celtis australis* subsp. *caucasica, Crataegus altaica, Juglans regia, Lonicera* sp., *Malus* sp., *M. domestica*, and *Salix* sp.), followed by *Stereum hirsutum* on 14 species of seven genera (*Juglans regia, Acer* sp., *A. tataricum* subsp. *semenovii, Salix iliensis, S. interior, S. alba, Fraxinus excelsior, Quercus* sp., *Q. robur, Celtis australis* subsp. *caucasica, Crataegus pseudoheterophylla* subsp. *turkestanica, Crataegus* sp., *Populus* sp., and *P. alba*), and *Trametes versicolor* on 11 species of nine genera (*Betula, Prunus, Crataegus, Lonicera, Quercus, Juglans, Populus, Malus*, and *Celtis*). Other wood-inhabiting species each colonized 1 to 10 plant species (Figure 10).

### The Distribution Range and Niches of Wood-Inhabiting Poroid and Corticioid Fungi in Uzbekistan

**FIGURE 11:**
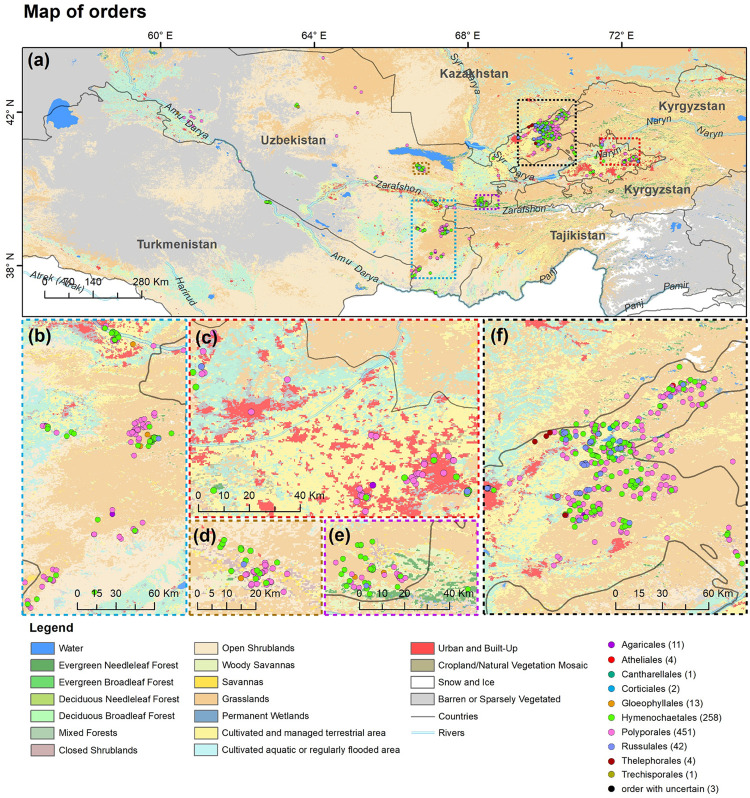
Spatial pattern of wood-inhabiting poroid and corticioid fungi at an order level based on 790 fungal records in **(a)** our study area, and five principal collecting areas shown zoom-in views in panels **(b–f)**.

By importing all known records of wood-inhabiting poroid and corticioid fungi in Uzbekistan into ArcGIS, a distribution GIS map of wood-inhabiting poroid and corticoid species in Uzbekistan was produced (Figure 11). The distribution of all records in the whole study area was first mapped (Figure 11a), and then five subareas where fungal records are relatively dense were presented (Figures 11b–f). Fungal species were most commonly collected in subarea **f** with 445 records in Western Tien Shan Mountains in Tashkent province, followed by subarea **b** with 142 records in Pamir-Alay Mountain in Samarkand, Qashqadaryo, and Surkhandaryo Provinces of Central and Southern Uzbekistan. Subareas **c**, **e**, and **d** have 58, 51, and 46 records, respectively, in Turkestan, Nurata, Kurama, and Fergana ranges in Pamir Alay and Western Tien Shan Mountains in Navoi, Jizzakh, and Fergana valley of Uzbekistan (Figures 11c–e).

Polyporales is the most commonly collected fungal order with 451 records in urban and mountain areas of Uzbekistan. The next most abundant order is Hymenochaetales with 258 records mainly distributed in Tashkent Botanical Garden, Chatkal Biospheric, Nurata, Zarafshan, Surkhan, and Hissar State Reserves in North-eastren and southern Uzbekistan. The orders Russulales (42), Gleophyllales (13), and Agaricales (11) are distributed in coniferous and deciduous mixed forest trees in Ugam, Chatkal, Turkestan, Pskem, Nurata, and Hissar ranges in Pamiy-Alay and Westren Tien Shan Mountains. Other orders with fewer records include Atheliales (4), Thelephorales (4), Cantharellales (1), and Trechisporales (1). In addition, three records have uncertain taxonomic positions at the order level. The most frequently recorded species in Uzbekistan are *Phellinus pomaceus* (34 records); *Stereum hirsutum* (27); *Trametes hirsuta* (25); *T. trogii* (22); *Lentinus tigrinus* (21); *Sanghuangporus lonicerinus* and *Trametes versicolor* (20 each); *Cerrena unicolor, Fomes fomentarius*, and *Inonotus hispidus* (17 each); *Bjerkandera adusta* and *Phellinus igniarius* (14 each); *Coriolopsis gallica, Fomitopsis betulina*, and *Trametes tephroleuca* (13 each); *Fuscoporia contigua* (12); *Phylloporia yuchengii* and *Tropicoporus linteus* (each 11); *Antrodia xantha, Cerioporus squamosus, Fomitiporia robusta*, and *Rigidoporus corticola* (each 10); and *Ceriporiopsis gilvescens* (8).

## Discussion

In this study, we compiled for the first time the species diversity of wood-inhabiting poroid and corticioid fungi in Uzbekistan. Comprehensive information of these species is provided, including taxonomic diversity, substrate preference, and distribution of geographic and landscape position, on the basis of 790 fungal records collected from 1950 to 2020.

A total of 153 wood-inhabiting poroid and corticioid species, belonging to 10 orders, 26 families, and 97 genera, were confirmed to be present mainly based on literature references, morphological examinations, and also on phylogenetic analysis wherever possible. Of these 153 species, 19 are new for mycobiota to Central Asia and 31 are reported for the first time in Uzbekistan. In addition, four taxa that may be new to science were discovered and must be examined further. The fungal diversity reported here for Uzbekistan is much lower than that in other regions where the diversity of wood-inhabiting poroid and corticoid fungi is well explored. For example, 1210 wood-inhabiting poroid and corticioid species, including ecologically similar hydnoid fungi, are recorded in China (Dai, 2011, 2012). Also, the number of wood-inhabiting poroid fungi recorded is 492 and 394 in North America and Europe, respectively (Ryvarden and Melo, 2014; Zhou et al., 2016). The low species number in Uzbekistan is partly due to the relatively small area, but also due to the lack of systematic field surveys and thorough identification with the aid of molecular sequencing. Few scientists study the ecologically important fungi of Uzbekistan. Although this report is the most comprehensive study of diversity of wood-inhabiting poroid and corticiod fungi in Uzbekistan today, it must be considered provisional. In contrast, tens of taxonomists have jointly contributed records of hundreds of (mainly new) wood-inhabiting poroid and corticioid species in China since the publications of Dai (2011, 2012). Therefore, even if we have reported the most comprehensive diversity of wood-inhabiting poroid and corticioid fungi in Uzbekistan to date, the current knowledge has to be considered as a provisional species checklist to be complemented. It is known that higher diversity of plant species results in a higher diversity of associated fungal species (Küffer and Senn-Irlet, 2005; Yamashita et al., 2010; Hawksworth and Lücking, 2017). Uzbekistan, with its mountainous landscape, is characterized by a high diversity of trees and shrubs, perhaps 500–600 species (Eastwood et al., 2009). Therefore, maybe more taxa of wood-inhabiting poroid and corticioid species, including new and even endemic species, still await to be revealed from Uzbekistan (Hyde et al., 2020; Yuan et al., 2020).

Most of the reported wood-inhabiting poroid and corticioid fungi in Uzbekistan are wood decomposers, which release matter and energy to the ecological system. These saprophytic species possess powerful enzymes, which can effectively degrade lignocellulose (Riley et al., 2014). In the current Uzbekistan mycota, several species in *Trametes* are considered to have potential biotechnological applications (Kneževič et al., 2013; Wang and Chen, 2019; Yang et al., 2020).

Although the resource recycling functions are generally considered beneficial to trees, forests, and humans, some of the wood-inhabiting poroid and corticioid fungi studied inhabit living trees as forest pathogens. According to previous studies (Kleyner, 1958; Sinadskiy and Bodartseva, 1960; Panfilova and Gaponenko, 1963; Sinadskiy, 1968; Khalikova, 1989; Baltaeva, 1992; Gafforov, 2014; Gafforov et al., 2014) and our field observations, *Inonotus hispidus, Bjerkandera adusta, Cerrena unicolor, Lentinus tigrinus, Fomes fomentarius, Laetiporus sulphureus, Phylloporia yuchengii* and *Ganoderma applanatum, Aurantiporus fissilis*, and some species from the genera *Trametes* and *Phellinus* can cause root rot disease. In addition, moreover, *Cerrena unicolor* also produces a stem canker of living *Acer semenovii, Juglans regia, Crataegus pseudoheterophylla* subsp. *Turkestanica*, and *Celtis australis* subsp. *caucasica*. Noteworthily, *Inonotus hispidus* is widespread in the walnut-fruit forests of Uzbekistan and damages up to 4% of the trees of *Juglans regia* and *Malus sieversii* trees in Baysun and Turkestan ranges of Pamir Alay Mountains (unpublished data Gafforov); *Phellinus igniarius* was observed both as a parasite and saprophyte of deciduous trees from the genera *Juglans, Salix*, and *Acer* in Ugam-Chatkal Natural State Park and Zaamin and Hissar State Reserve. The forest diseases caused by these wood-inhabiting poroid and corticioid fungi and the corresponding economic loss should be considered by relevant management departments.

In addition, some macrofungi including wood-inhabiting poroid and corticioid species are important edible and medicinal fungi (Wu et al., 2019; Zhou et al., 2020). Some poroid species known from Uzbekistan are recognized as valuable medicinal fungi, while *Grifola frondosa, Laetiporus sulphureus*, and *Sarcodon imbricatus* are important edible fungi. Cultivation of wood-inhabiting fungi is an important agricultural industry worldwide, especially in East Asia. Several medicinal and edible species, like *Ganoderma* spp. and *Auricularia* spp. in mainland China, *Taiwanofungus* in Taiwan, China, and *Sanghuangporus* spp. in South Korea, have huge economic value. In China, edible and medicinal fungi are the fifth largest crop industry (Dong et al., 2017). Industrial development of suitable endemic wood-inhabiting poroid and corticioid fungi will undoubtfully benefit the Uzbekistan economy. Uzbekistan materials must be directly studied for utilization of these fungal resources. For this purpose, strains of wood-inhabiting poroid and corticioid fungi firstly need to be isolated and preserved in public organizations.

The proportion of wood-inhabiting poroid and corticioid fungi found on different hosts differs in Uzbekistan from that in comparable temperate and warm temperate forest zones of China (Zhou et al., 2011). Compared with the proportions in temperate and warm temperate forest zone (Zhou et al., 2011), the proportion of wood-inhabiting poroid and corticioid species in Uzbekistan found on deciduous wood (72.46%) is similar, but the proportion on coniferous wood is much higher (23.91%) and that on both groups of wood is much lower (3.62%). The differences in the proportions on coniferous wood and both groups of wood compared to the Chinese case may be either the real status in Uzbekistan or a misleading failure to observe that some species recorded exclusively on coniferous wood do actually occur also on deciduous wood. Many more field surveys are needed to clarify this issue.

Among the 100 woody plant species belonging to 23 families and 42 genera recorded as hosts for wood-inhabiting poroid and corticioid species, Salicaceae, Pinaceae, and Rosaceae are the most favored, and *Populus, Quercus, Juglans*, and *Salix* are the most favored host genera.

To well understand the spatial patterns of wood-inhabiting poroid and corticioid fungi, their distributions associated with geography and landscapes were visualized using GIS maps (Figure 11). Wood-inhabiting poroid and corticioid species in Uzbekistan are distributed mostly in the regions of open shrublands and grasslands, and rarely in various kinds of forests. This distribution pattern is opposite to the natural habitats of such fungi as reported in previous studies. For example, Zhou and Dai (2012) reported that reserved forest with amounts of woody substrates for the growth of wood-inhabiting poroid fungi has significantly higher polypore diversity than unprotected forest. Moreover, normally, forests have higher species diversity of woody plants, which result in higher diversity of wood-inhabiting poroid fungi (Dai et al., 2015). This unusual phenomenon in Uzbekistan might be caused by a lower proportion of field surveys carried out in virginal forests due to difficult access. Therefore, more efforts should be made to reveal the diversity of wood-inhabiting poroid and corticioid fungi in the most primeval forests.

The two fungal orders with highest record diversity, viz., Polyporales and Hymenochaetales, are common in the whole studied area (Figure 11a). This reflects that geography and landscape factors do not have significant effects on the distribution of these two fungal orders. These two fungal orders have high species diversity (Table 2), resulting from a wide adaption to the environment. Species diversity of these two orders are also highest in other regions of the world (Dai, 2012; Ryvarden and Melo, 2014; Zhou et al., 2016). Russulales, the order with the third highest recorded diversity, also occurs in all five subareas, but mainly in the subarea **f**, viz., Western Tien Shan Mountains in Tashkent region (Figure 11f). Other orders and the species without a confirmed position at the order level are present only in small areas. Such species may be sensitive to environmental changes. So, to sustain species diversity of wood-inhabiting poroid and corticioid fungi, special attention should be paid to protecting their habitats. Using the GIS data, the potential future distribution of certain important (biotechnological, pathogenic, medicinal, and edible) wood-inhabiting poroid and corticioid species could be predicted. Similar studies have been performed on some wood-inhabiting poroid and corticioid species worldwide (Yuan et al., 2015; Elias et al., 2020). Generally, knowledge of species diversity and occurrence in an area is a baseline for benefiting from ecosystem services, monitoring environmental changes, and implementing conservation actions (Mueller and Schmit, 2007; Hibbett et al., 2007, 2011; Brock et al., 2009; Hyde et al., 2013; Osmundson et al., 2013; Truong et al., 2017). Therefore, the current GIS data are important for management and utilization of wood-inhabiting poroid and corticioid fungi in Uzbekistan.

In conclusion, this study provides the first comprehensive, thoroughly annotated checklist of species diversity of wood-inhabiting poroid and corticioid fungi in Uzbekistan. These species are ecologically and economically important as decomposers, pathogens, and sources of food and medicines. Beyond local scale, these data are also crucial as a supplement of the global knowledge of wood-inhabiting poroid and corticioid fungi and for elucidating the evolutionary history of wood-inhabiting poroid and corticioid fungi worldwide. More importantly, the current project exploring wood-inhabiting poroid and corticioid fungi in a less studied country may initiate similar explorations in other Central Asian countries and also other regions worldwide, which will largely fulfill the knowledge gap of wood-inhabiting poroid and corticioid fungi in certain rarely studied regions.

## Data Availability Statement

The datasets presented in this study can be found in online repositories. The names of the repository/repositories and accession number(s) can be found in the article/supplementary material.

## Author Contributions

YG and MY collected fungal specimens and performed DNA lab work. YG and AO were responsible for morphological identification and management of collection data. L-WZ and YG performed the molecular phylogenetic analyses. YZ mapped the fungal taxa. YG, AO, and L-WZ drafted the manuscript. EL, AG, DS, LP, and LC improved and revised it. All authors have read the final manuscript version and approved it. All authors contributed to the article and approved the submitted version.

## Conflict of Interest

The authors declare that the research was conducted in the absence of any commercial or financial relationships that could be construed as a potential conflict of interest.

## Footnotes

1 http://www.treebase.org

2 http://tree.bio.ed.ac.uk/software/tracer/

3 https://dwc.tdwg.org

4 https://doi.org/10.15468/vsru5z

5 https://gbif.univer.kharkov.ua/resource?r=aphyllo_uz

6 google.com/earth/versions/
